# Conformational flexibility within the nascent polypeptide–associated complex enables its interactions with structurally diverse client proteins

**DOI:** 10.1074/jbc.RA117.001568

**Published:** 2018-04-12

**Authors:** Esther M. Martin, Matthew P. Jackson, Martin Gamerdinger, Karina Gense, Theodoros K. Karamonos, Julia R. Humes, Elke Deuerling, Alison E. Ashcroft, Sheena E. Radford

**Affiliations:** From the ‡ Astbury Centre for Structural Molecular Biology, School of Molecular and Cellular Biology, Faculty of Biological Sciences, University of Leeds, Leeds LS2 9JT, United Kingdom and; the § Department of Biology, Institute of Molecular Microbiology, University of Konstanz, 78454 Konstanz, Germany

**Keywords:** protein folding, protein misfolding, protein cross-linking, chaperone, molecular chaperone, NAC native mass spectrometry (MS), nuclear magnetic resonance (NMR)

## Abstract

As newly synthesized polypeptides emerge from the ribosome, it is crucial that they fold correctly. To prevent premature aggregation, nascent chains interact with chaperones that facilitate folding or prevent misfolding until protein synthesis is complete. Nascent polypeptide–associated complex (NAC) is a ribosome-associated chaperone that is important for protein homeostasis. However, how NAC binds its substrates remains unclear. Using native electrospray ionization MS (ESI-MS), limited proteolysis, NMR, and cross-linking, we analyzed the conformational properties of NAC from *Caenorhabditis elegans* and studied its ability to bind proteins in different conformational states. Our results revealed that NAC adopts an array of compact and expanded conformations and binds weakly to client proteins that are unfolded, folded, or intrinsically disordered, suggestive of broad substrate compatibility. Of note, we found that this weak binding retards aggregation of the intrinsically disordered protein α-synuclein both *in vitro* and *in vivo*. These findings provide critical insights into the structure and function of NAC. Specifically, they reveal the ability of NAC to exploit its conformational plasticity to bind a repertoire of substrates with unrelated sequences and structures, independently of actively translating ribosomes.

## Introduction

Upon emerging from the ribosome, most proteins have to fold into a unique three-dimensional structure to become biologically active. Finding the correct fold and avoiding misfolding and aggregation are major problems for polypeptide chains in the crowded cellular environment. This problem is exacerbated by the slow rate of synthesis compared with the rapid rate of folding of most proteins, which provides an opportunity for partially synthesized proteins to become trapped in misfolded or aggregation-prone states (1–3). Help is at hand by molecular chaperones: essential proteins found across all kingdoms of life that facilitate protein folding, unfolding, and degradation, forming a network of interactions necessary for maintaining a healthy proteome (1). Some chaperones have the specific role of interacting with newly synthesized polypeptides to prevent intermolecular interactions that could result in aggregation (4–6). Two general groups of chaperones exist: those that interact with the ribosome and the nascent chain simultaneously, thereby controlling the early stages of folding (7), and those that are not involved until the complete polypeptide has emerged from the ribosome exit tunnel (8).

Ribosome-associated chaperones vary across species. Bacteria possess the well-characterized trigger factor, a chaperone with a molecular cradle structure that captures polypeptide chains as they emerge from the ribosome (9, 10). Its interior structure is lined with hydrophobic residues that provide multiple contact points for its unfolded protein substrates (11). Within eukaryotes, the chaperone network is more complex and consists of two systems: the ribosome-associated complex (12, 13) and the nascent polypeptide–associated complex (NAC)^6^ (14, 15).

**Figure 1.**
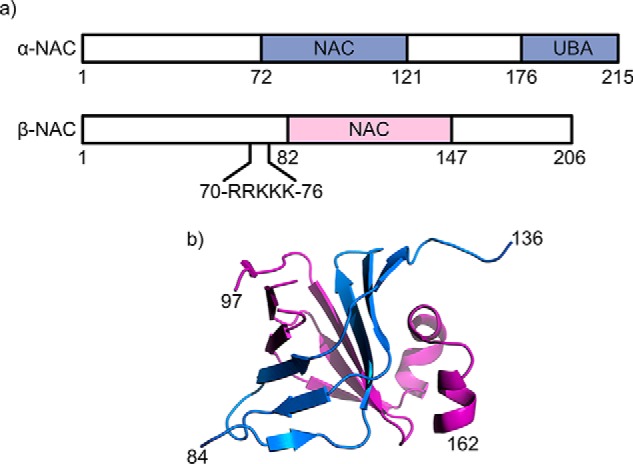
**Schematic representation of NAC.** *a,* domain structure of human NAC highlighting the UBA domain on α-NAC and the location of the ribosome-binding motif (RRK(*X*)*_n_*)KK) on β-NAC. *b,* dimerization of the NAC domains, with α-NAC shown in *blue* and β-NAC shown in *purple* (PDB 3LKX) (18).

Whereas NAC is highly conserved across eukaryotes, archaea possess a NAC homodimer consisting of two α-subunits, and yeast and mammalia possess a heterodimer formed of α- and β-NAC subunits (16). The overall sequence homology between the 215-residue α-NAC and the 206-residue β-NAC subunits is only 26%. However, both subunits contain a highly conserved (41% sequence identity) central NAC domain of ∼61 residues (Fig. 1*a*). X-ray crystal structures of truncated human NAC (Fig. 1*b*) show that the α- and β-NAC subunits associate through their six-stranded β-barrel–like central NAC domains, although the structure, function, and interactions made by the N- and C-terminal domains of the α-NAC and β-NAC subunits remain unknown (17, 18). Human heterodimeric NAC has been shown to be more stable than its homodimeric assemblies, and importantly, homodimers of α-NAC do not form in the presence of β-NAC, suggestive of a specific heterodimeric complex despite the close sequence homology of the central NAC domains (17).

α-NAC differs from β-NAC in that it contains a single ubiquitin-associated (UBA) domain that comprises the C-terminal 40 amino acids of the polypeptide chain (Fig. 1*a*) (19). Although the role of this domain remains unclear, a NAC variant that lacks the UBA domain has been shown to be a more potent suppressor of protein aggregation *in vivo* suggesting a regulatory role for the UBA domain in the chaperone activity of the complex (20). Previous reports have also shown that NAC interacts with translating ribosomes reversibly in a 1:1 stoichiometry, and although α-NAC forms contacts with both the ribosome and the nascent chain, it is the β-NAC subunit that mediates the dynamic interaction with the ribosome via the –RRKKK– motif (residues 71–75 (Fig. 1*a*)) in its N-terminal region (21–23). Previous studies have also shown that NAC is able to protect the emerging nascent chain from proteolysis, confirming its role in guarding nascent chains during their synthesis (24). Multiple functions have been suggested for NAC *in vivo*, including protection of nascent chains from proteolysis and regulation of apoptosis, and there is also evidence of a homodimer of α-NAC bound to DNA and RNA, implying that NAC can take the role of a transcription factor (16). Recently, NAC depletion in *Caenorhabditis elegans* was shown to cause mistargeting of translating ribosomes to the endoplasmic reticulum (ER) membrane and mistranslocation of mitochondrial proteins into the ER. Loss of NAC activity also reduced the median life span of *C. elegans* by 10 days by means of inducing ER and mitochondrial stress (25). Despite its ubiquity and central importance in protein synthesis and folding, how NAC binds and chaperones its substrate proteins, however, remained unknown.

Over the past 20 years, native MS has become a powerful tool for the interrogation of noncovalent protein complexes and their interactions (26–31). Complementary information can be obtained using hydrogen-exchange MS (32) and fast photochemical–oxidation of proteins (33), each combined with identification of the sites of modification using proteolysis followed by LC-MS/MS. Furthermore, native MS methods enable the structure, stability, and conformation of proteins and their complexes to be investigated directly upon their ionization into the gas phase (34–36) With the advancement of instruments and software, it has also become possible to interrogate transient noncovalent interactions between proteins using MS-based techniques. For example, chemical cross-linking can be used to provide residue-specific information about transiently interacting partners by sequencing of cross-linked products using LC-MS/MS (37).

Here, we use ESI-MS combined with ion-mobility spectrometry–MS (ESI-IMS-MS) to study the conformational properties of wildtype (WT) α/β-NAC (WT-NAC) from *C. elegans* for the first time. We also use MS-based techniques to provide information about the structure, dynamics, and interactions of WT-NAC with potential substrates in different conformational states. In addition, the conformations of WT-NAC and the heterodimer lacking the UBA domain (ΔUBA-NAC) are compared using ESI-IMS-MS to establish the effect of the UBA domain on conformational properties of NAC. It has been suggested that the UBA domain, which is attached to the α-NAC subunit via a highly flexible 34-residue linker, may regulate the chaperone activity of NAC (20). In parallel, chemical cross-linking of complexes formed between WT-NAC and α-synuclein (a 140-residue intrinsically disordered protein (IDP)) (38, 39) and the 87-residue bacterial immunity protein Im7, in its four helical native state and unfolded by creating the triple mutant (TM Im7) L18A/L19A/L37A (40, 41), were used to compare the interactions of WT-NAC with different protein substrates and to identify their binding sites. Combined with analysis of the NAC–α-synuclein complex using ^1^H-^15^N NMR, and assays of the effect of NAC binding on α-synuclein aggregation *in vitro* and *in vivo*, the results reveal that NAC binds substrates with very different sequence and structural properties, forming weak interactions in a dynamic complex independent of the presence of the UBA domain or actively translating ribosomes.

## Results

### ESI-MS reveals both compact and extended conformations of NAC

**Figure 2.**
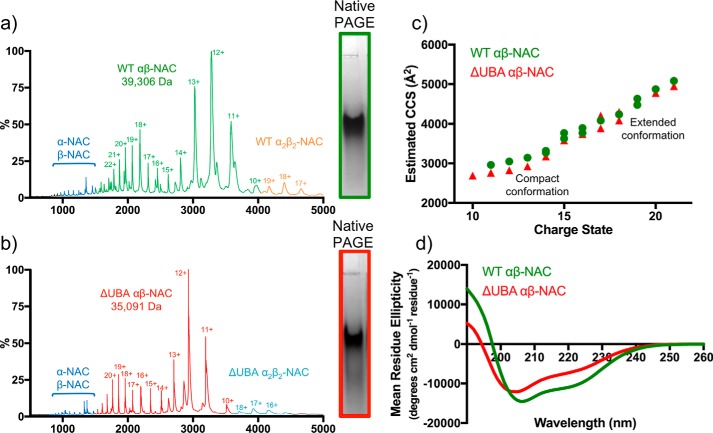
**Comparison of native ESI mass spectra of WT-NAC and ΔUBA-NAC.** *a* and *b*, native ESI mass spectra of WT-NAC (*a*) and ΔUBA-NAC (*b*). Each spectrum shows two charge-state distributions for each heterodimer (*green* and *red* for WT-NAC and ΔUBA-NAC, respectively), with low populations of α_2_β_2_-NAC dimers as indicated. In addition, low populations of dissociated α-NAC and β-NAC subunits are observed (*blue*). The *insets* show the proteins analyzed by native PAGE, which reveal a single band of the heterodimer and no evidence of dissociation. *c,* estimated collision cross-sections (*CCS*) from ESI-IMS-MS experiments for WT-NAC (*green circles*) and ΔUBA-NAC (*red triangles*) show that compact and extended forms co-exist for both species (see also Table S1). *d,* far-UV CD spectra of WT-NAC (*green*) and ΔUBA-NAC (*red*). The secondary structure content obtained using CONTIN (58) is given in Table S2.

The native ESI mass spectrum of purified *C. elegans* NAC obtained in 100 mm ammonium acetate buffer at pH 6.9 is shown in Fig. 2*a*. The spectrum confirms the presence of a heterodimer of α-NAC and β-NAC with a molecular mass of 39,306 Da (theoretical mass = 39,304 Da). The spectrum shows the presence of at least two distinct protein conformations for the α/β-NAC dimer: the most abundant species has 11^+^ to 13^+^ charges and is likely the most native-like conformer, with a second population being more highly charged (14^+^ to 22^+^), suggestive of more expanded conformations. NAC dimers (a_2_β_2_) and a small amount of the free α-subunit (measured mass = 21,803 Da; theoretical mass = 21,802 Da) and β-subunit (measured mass = 17,504 Da; theoretical mass = 17,502 Da) were also visible in the spectrum. Control experiments using native PAGE showed only a single band (*inset* of Fig. 2*a*) suggesting that some dissociation of the heterodimer alongside self-association to larger species occurs in the gas phase but does not occur in solution. Interestingly, native ESI-MS of the NAC construct lacking its UBA domain (ΔUBA-NAC, measured mass = 35,091 Da; theoretical mass = 35,091 Da) showed the same distribution of charge states as WT-NAC, with the 12^+^ charge state ions being the most abundant species (Fig. 2*b*), ruling out major conformational changes of the protein upon deletion of this domain.

Native ESI-IMS-MS confirmed that species that have a range of compact and extended conformers of both WT-NAC and ΔUBA-NAC exist in the gas phase (Fig. 2*c*, Table S1). The measured collision cross-sections (CCS) for the lowest observed (11^+^) charge states were 2962 ± 88 and 2761 ± 83 Å^2^ for WT-NAC and ΔUBA-NAC, respectively (Fig. 2*c*). The function of the UBA domain within NAC has yet to be ascertained, but we hypothesized that the extended highly charged conformers of WT-NAC observed here using ESI-MS could result from the attachment of the UBA domain to the NAC core via a flexible linker (Fig. 1*a*). The ESI-IMS-MS results presented here rule out such a hypothesis and show instead that the extended conformation observed for WT-NAC is also observed for the protein lacking the UBA domain. Importantly, the finding that the difference in CCS of WT-NAC and ΔUBA-NAC is only ∼200 Å^2^, consistent with the expected CCS of the folded UBA domain, confirms that NAC remains folded in the gas phase despite the lack of bulk solvent water (42, 43).

CD spectroscopy showed that WT-NAC contains 33% disorder, 27% helical, and 17% β-stranded structure (Fig. 2*d* and Table S1). Deletion of the 35-residue UBA domain resulted in a shift in the major peak from 206 to 204 nm, and the overall α-helical content was reduced to ∼15%. This indicates the UBA domain makes a large contribution to the total α-helical content of NAC, consistent with the known predominantly helical structure of the UBA domain (44). The proportion of unstructured protein was increased to 36% in this variant compared with WT-NAC (Table S2).

### CIU indicates ΔUBA-NAC is more susceptible to unfolding than WT-NAC

**Figure 3.**
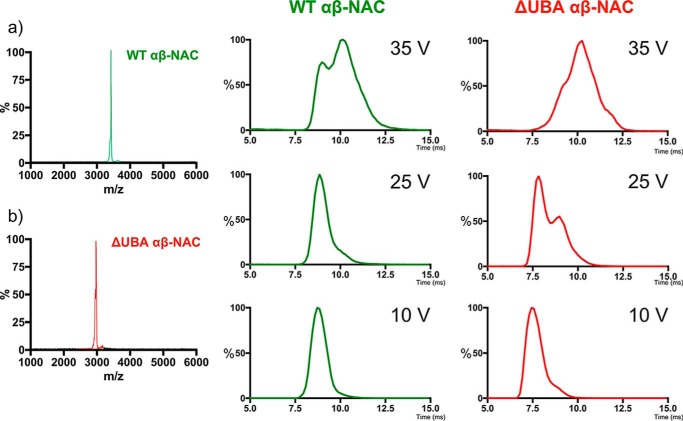
**Collision-induced unfolding (CIU)-IMS-MS for the 12^+^ charge state ions of WT-NAC and ΔUBA-NAC.** The quadrupole selected 12^+^ charge state ions are shown at a trap collision energy of 25 V for WT-NAC (*a*) andΔUBA-NAC (*left*) (*b*). Extracted ATDs for these charge states at 10 V, 25 V and 35 V are shown alongside.

To compare the stability of the WT-NAC and ΔUBA-NAC heterodimers in more detail, the proteins were each examined using CIU. The 12^+^ charge state ions for WT-NAC (Fig. 3*a*) and ΔUBA-NAC (Fig. 3*b*) were each isolated in the first (quadrupole) analyzer of the ESI-MS-MS mass spectrometer, and the collision energy increased stepwise in the trap cell prior to the IMS cell and second (TOF) analyzer. No dissociation of the heterodimers was observed at 10 V with drift times of 8.7 and 7.4 ms, respectively (Fig. 3, *lower panels*). Clear differences in the unfolding patterns of the two proteins were observed, however, as the collision voltage was increased. For WT-NAC, a single conformation persisted at 25 V, with a second, more unfolded conformer (drift time 10.3 ms) becoming populated at 35 V. By contrast, ΔUBA-NAC showed evidence for substantial unfolding at 25 V, with the appearance of a second population (∼40% of molecules) with an arrival time of 9.1 ms in the IMS drift-time plot. Finally, at 35 V ΔUBA-NAC shows significant conformational rearrangements, with little, if any, residual population of native-like species with a drift time 7.8 ms. Instead, a broad arrival time distribution containing more highly expanded species is observed (Fig. 3, *top panel*). The results presented here using far-UV CD, ESI-IMS-MS, and CIU together show that deletion of the UBA domain does not perturb the structure of the NAC heterodimer but results in a complex that is more susceptible to unfolding in the dimeric state. This may result in a complex more able to bind its substrates, consistent with *in vivo* observations of an increased chaperone capacity of NAC upon deletion of the UBA domain (20).

### Limited proteolysis of WT-NAC and ΔUBA-NAC

**Figure 4.**
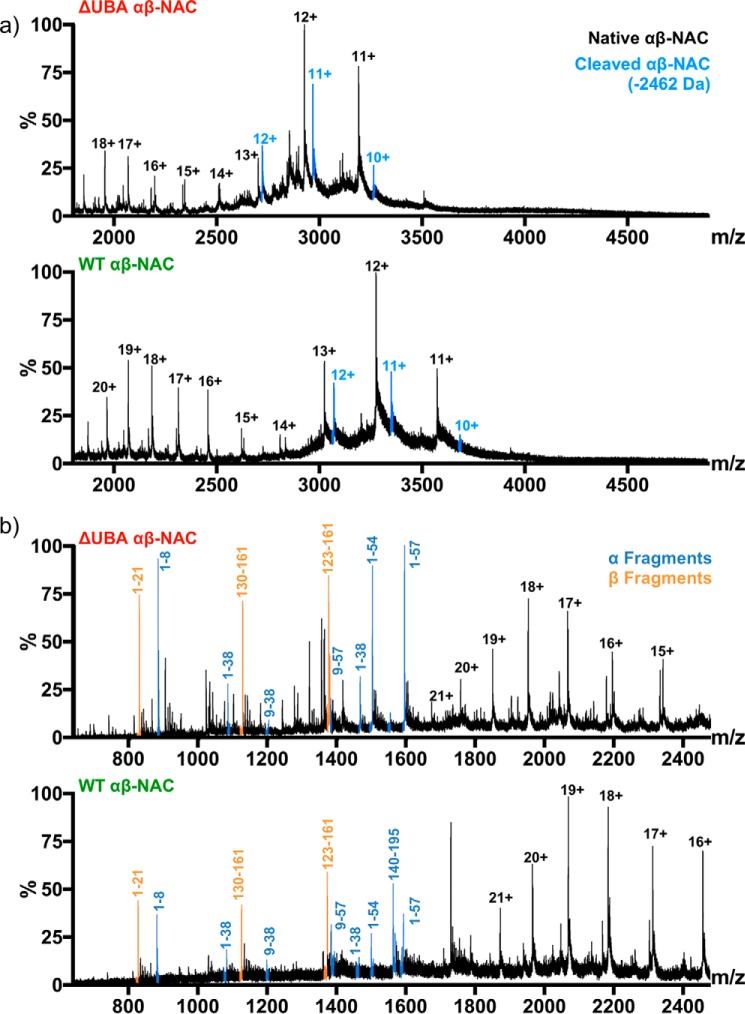
**Limited proteolysis of WT-NAC and ΔUBA-NAC followed by ESI-MS analysis.** Each protein was treated with 1:500 (w/w) trypsin/substrate for 15 min at room temperature. *a,* native ESI-MS shows a truncated complex with a reduced mass of 2462 Da (*blue peaks*) and, which remains assembled, is the first cleavage product for both WT-NAC and ΔUBA-NAC. *b,* tryptic fragments observed in the low *m/z* range reveal a range of peptides released from the N-terminal domain of α-NAC (*blue*) and from the C-terminal domain of β-NAC (*orange*). The same fragments are observed for WT-NAC and ΔUBA-NAC. *Numbers* denote the fragments within each NAC domain. Charge states resulting from the intact (undigested) NAC are shown in *black*.

Limited proteolysis followed by MS analysis of WT-NAC and ΔUBA-NAC was next carried out to investigate whether the presence of the UBA domain influences the accessibility of the α- and β-domains to protease (Fig. 4, *a* and *b*). After incubation of NAC with trypsin (1:500 (w/w) trypsin/NAC) for 15 min at 20 °C, a cleaved protein with a mass 2462 Da less than the native protein was observed (Fig. 4*a*, *lower panel*). The population of this species became more intense relative to uncleaved NAC over time (data not shown). This mass loss from the native protein is consistent with possible loss of MTGSTETRQKEVK (α-NAC residues 1–13), ADEQ (α-NAC residues 158–161), and MDSK (β-NAC residues 1–4) or MTGSTETRQKEVK (α-NAC residues 1–13), and NETKADEQ (β-NAC residues 154–161). Notably, the same mass loss was also detected in the ΔUBA-NAC sample (Fig. 4*a*, *upper panel*), indicating that these sequences are similarly accessible to protease in both heterodimers. In the low *m/z* range of the spectrum (*m/z* 800–1600), fragments corresponding to peptides from the N-terminal region of α-NAC (*e.g.* 1–38, 9–38, and 9–57 in WT α-NAC and 1–38, 1–54, and 2–57 of ΔUBA-NAC) and the C-terminal region of β-NAC (130–161 and 123–161 in WT-NAC and ΔUBA-NAC, respectively) were most abundant (Fig. 4*b*). This indicates that these regions of the protein are most accessible to protease, whereas the core NAC regions, which form the dimer interface, remain uncleaved, as expected from their folded structure. These observations are consistent with the known domain architecture of the NAC complex (Fig. 1*a*) and show that the UBA domain does not alter the accessibility of the protein complex to trypsin, although deletion of this domain decreases the stability of the complex to collision-induced unfolding.

### Mapping NAC substrate interactions

**Figure 5.**
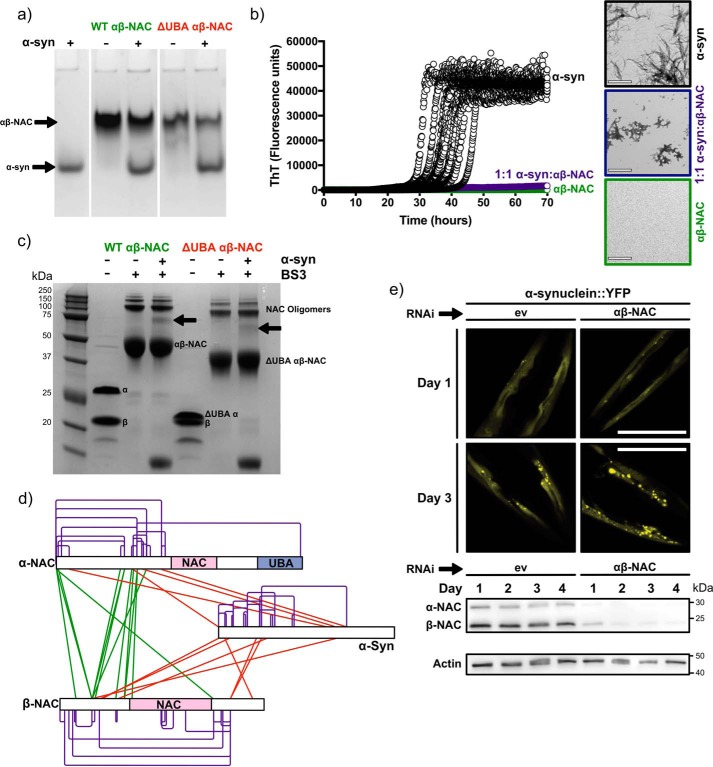
**Interactions of α-synuclein with WT-NAC and ΔUBA-NAC.** *a,* Coomassie Blue-stained native polyacrylamide gel of α-synuclein alone and mixed with an equimolar concentration of WT-NAC or ΔUBA-NAC, showing that a stable complex is not detected. *b,* kinetic aggregation assays of α-synuclein (125 μm, *black*), a 1:1 molar ratio of α-synuclein with WT-NAC (*purple*), and WT-NAC alone (*green*) measured using ThT fluorescence. An equimolar concentration of WT-NAC to α-synuclein inhibits its aggregation over a 70-h time scale. Images alongside show negative stain transmission electron micrographs of the reaction end point (at 70 h) for each sample. *Scale bar,* 500 nm. *c,* SDS-PAGE analysis of cross-linked WT-NAC and ΔUBA-NAC mixed with equimolar α-synuclein (a 50-fold excess of BS3 was used (“Experimental procedures”)). The *arrows* highlight the covalent complex between NAC and α-synuclein. *d,* map of cross-links between WT-NAC and α-synuclein identified following in-gel tryptic digestion of the *band arrowed* in *c*. Intra-NAC or intra-α-synuclein cross-links (*purple*), inter-NAC cross-links (*green*), and NAC–α-synuclein cross-links (*red*) are shown. Peptides identified are listed in Tables S3–S5. *e,* RNAi-mediated NAC depletion leads to increased α-synuclein puncta formation *in vivo*. Fluorescence microscope images of transgenic worms (head regions are shown) expressing α-synuclein::YFP in body-wall muscle cells. Worms were grown on empty vector control (*ev*) or αβ-NAC RNAi, respectively. Images were taken at days 1 and 3 of adulthood. *Scale bar,* 50 μm. *Inset,* Western blotting shows NAC protein expression levels, at indicated time points, by immunoblotting. Immunoblot against actin served as loading control.

To identify residues required for substrate binding, WT-NAC and ΔUBA-NAC were each mixed in a 1:1 molar ratio with α-synuclein (a 140-amino acid IDP) (39), which was used as a model for unfolded polypeptide substrates as they may emerge from the ribosome. Despite exploring a wide range of instrumental conditions (increased backing pressure, increased trap cell pressure, and decreased activation voltages), a complex was not observed using native ESI-MS. No complex was observed via native PAGE (Fig. 5*a*). However, incubating α-synuclein in conditions in which it is aggregation-prone (125 μm protein, Dulbecco's PBS, 600 rpm agitation), with an equimolar concentration of WT-NAC, prevented α-synuclein aggregation, at least over a time scale of 70 h (Fig. 5*b*), suggesting that the proteins form a weak complex that cannot be maintained in the gas phase or on a native polyacrylamide gel, but it is sufficient to have a dramatic and protective effect on protein aggregation. To probe this NAC–client interaction further, WT-NAC and α-synuclein were mixed and cross-linked using the homobifunctional amine cross-linker, BS3 (see “Experimental procedures”), and the samples were examined by SDS-PAGE to search for cross-linked products. Upon cross-linking WT-NAC alone, a band with the mobility expected for a cross-linked NAC αβ dimer was observed (∼45 kDa) (Fig. 5*c*), consistent with the theoretical mass of a 1:1 αβ-NAC dimer of ∼39 kDa. Weaker bands corresponding to NAC dimers (α_2_β_2_, at ∼100 kDa), trimers (α_3_β_3_, at ∼150 kDa), and tetramers (α_4_β_4_, at ∼200 kDa) were also visible (Fig. 5*c*, *leftmost lanes*). Similar products were observed for ΔUBA-NAC at lower molecular mass, consistent with deletion of the 4.2-kDa UBA domain (Fig. 5*c*, *rightmost lanes*). Immediately following cross-linking, WT-NAC was also buffer-exchanged into 100 mm ammonium acetate, pH 6.9, and the sample was analyzed by native ESI-MS. Interestingly, a significant decrease in the intensity of the charge state distribution for the extended conformation of NAC was observed in this sample, suggesting that the cross-linking has stabilized flexible regions of NAC such that they are no longer able to acquire as many positive charges (Fig. S1).

**Figure 6.**
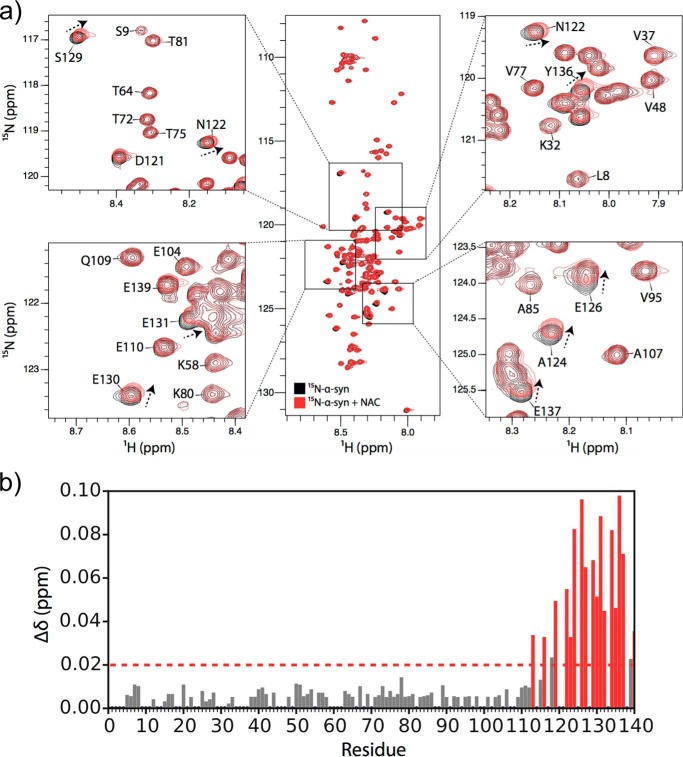
**^1^H–^15^N HSQC spectra of α-synuclein following addition of WT-NAC.** *a,* spectra are overlaid of 50 μm ^15^N-labeled α-synuclein alone (*black*) and in the presence of 1 molar eq of WT-NAC (*red*). Resonances that shift upon the addition of NAC are indicated with an *arrow. b,* chemical shift perturbations in α-synuclein upon NAC binding. Residues exhibiting a significant chemical shift difference (>1 S.D. over the mean (*dashed line*)) are highlighted in *red*.

Cross-linking α-synuclein and NAC mixed in a 1:1 molar ratio resulted in a new band observed by SDS-PAGE with a mobility of ∼70 kDa (Fig. 5*c*, *arrow*) consistent with a 1:1 NAC–α-synuclein complex. Importantly, there was no evidence for higher molecular weight complexes or complexes involving >1 α-synuclein bound to WT-NAC or ΔUBA-NAC, indicating that a weak but specific complex was formed. Consistent with this observation, ^1^H–^15^N HSQC spectra of ^15^N-labeled α-synuclein in the presence or absence of 1 molar eq of WT-NAC showed small but significant chemical shift perturbations for residues in the C-terminal ∼25 residues of the protein (Fig. 6, *a* and *b*), suggestive that a specific, weak complex had formed. The C-terminal region of α-synuclein is highly acidic, suggesting that electrostatic interactions between this region of α-synuclein and basic residues in NAC form at least part of the interaction interface. Notably, a similar complex was formed, albeit at lower molecular weight, when α-synuclein was mixed with the ΔUBA-NAC (Fig. 5*c*, *arrow*), indicating that loss of the UBA domain does not preclude NAC from binding α-synuclein. The UBA domain, therefore, is not crucial for α-synuclein binding.

To identify residues involved in forming the NAC–α-synuclein complex, an in-gel tryptic digest of the band arising from the putative complex was performed, and LC-MS/MS was used to identify lysine residues that form intra- or intermolecular cross-links (Fig. 5*d* and Tables S3–S5). Many intermolecular cross-links were observed between α-NAC and β-NAC (Table S3), especially involving their N-terminal domains (Fig. 5*d*), suggestive of many, possibly transient, interactions between these regions. Only one cross-link was observed between the N- and C-terminal domains of α-NAC, consistent with this complex being extended in nature with few interactions with the UBA domain (Fig. 5*d*). Importantly, of the eight lysines present in the αβ-NAC core, only one intramolecular cross-link was observed between Lys-84/Lys-86 in α-NAC, consistent with the known crystal structure of the α/β-NAC core (PDB 3LKX) (18). Lys-72 within α-NAC cross-linked to the N terminus of α-NAC, consistent with the known dynamics in this region. Furthermore, Lys-82 within β-NAC reacted with BS3 but did not form cross-links, suggesting that this amino acid is solvent-accessible but does not interact with client proteins or the adjacent lysine-rich ribosome-binding motif within β-NAC (Tables S3 and S6). These data are consistent with the view that the cross-links observed reflect specific interactions formed within the α/β NAC complex (Fig. 5*d*). Interestingly, several cross-links were observed between the N- and C-terminal domains of β-NAC, suggesting that this subunit is more compact than the α-subunit in the NAC–substrate complex. An array of cross-links was observed between the N- and C-terminal domains of NAC and residues 1–102 of α-synuclein (Table S5). This interaction must also involve the acidic C-terminal region of α-synuclein revealed by NMR chemical shift perturbation (Fig. 6*b*), but since this region lacks lysines, no cross-links were observed. The absence of chemical shift perturbation in other regions of α-synuclein that do form cross-links to NAC is consistent with a diffuse binding interface in which the side-chain ϵ-NH_3_^+^ of lysine residues form transient, presumably electrostatic interactions with NAC, without perturbation of the chemical environment of the main chain, reminiscent of the binding of other ATP-independent chaperones with their clients (9, 45). Together, the results highlight the synergy of the MS and NMR approaches taken and confirm that NAC binds weakly to α-synuclein, forming transient interactions that are able to suppress its aggregation.

To address the physiological relevance of our finding that NAC is able to suppress α-synuclein aggregation *in vitro*, we used a transgenic *C. elegans* strain expressing α-synuclein fused to yellow fluorescent protein (Fig. 5*e*). By day 3 of adulthood, these animals showed α-synuclein::YFP puncta. Strikingly, depletion of NAC by RNAi increased the number of α-synuclein puncta, showing that the presence of NAC also ameliorates α-synuclein aggregation *in vivo*.

### Does NAC interact with folded proteins?

**Figure 7.**
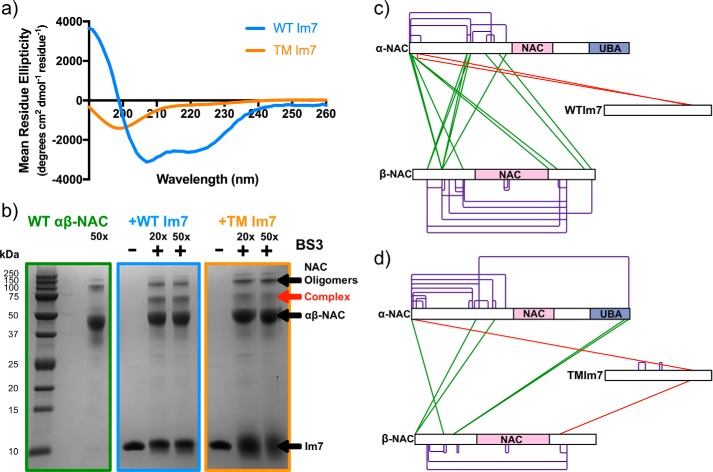
**NAC binds WT Im7 and TM Im7.** *a,* far-UV CD spectra of WT Im7 (*blue*) and TM Im7 (*orange*) under the conditions used for the cross-linking experiments (see “Experimental procedures”). *b,* SDS-PAGE analysis of NAC cross-linked alone (*green*) or to WT Im7 (*blue*) or TM Im7 (*orange*) using BS3. *Lanes* show the addition of a 20 or 50× excess of BS3. The *red arrow* highlights the putative complex between NAC and the protein substrates. *c,* map of the cross-links identified following in-gel tryptic digest of the putative NAC–Im7 complex: intra-NAC cross-links (*purple*), inter-NAC cross-links (*green*), and NAC-WT Im7 cross-links (*red*). *d,* map of the cross-links identified following in-gel tryptic digest of the putative NAC–TM Im7 complex band colored as in *c*. Peptides identified are listed in Tables S6–S10.

As NAC was shown to interact with an IDP, we next used native ESI-MS and chemical cross-linking to determine whether NAC can also bind folded proteins. For this, the 87-residue, four-helical bacterial immunity protein Im7 was used (40), alongside its triple mutant (L18A/L19A/L37A; TM Im7), which is trapped in an unfolded state (Fig. 7*a*) (41). Im7 has been shown previously to fold in seconds (40) and hence could be considered as a mimic of a protein domain that folds rapidly upon emergence from the ribosome tunnel. By contrast, TM Im7 was used as a model for an unfolded chain with an amino acid composition distinct from that of a highly charged and poorly hydrophobic IDP (46). Notably, both Im7 and TM Im7 have been shown previously to bind the ATP-independent chaperone Spy (45).

Cross-linking WT Im7 or TM Im7 to NAC resulted in a unique band at ∼60 kDa when analyzed using SDS-PAGE suggesting that NAC can bind to Im7 in both its folded and unfolded states (Fig. 7*b*). No complex was observed using native ESI-MS (data not shown) indicating that the complex is too lowly populated, or too weak to survive passage into the gas phase, as was also observed when NAC was mixed with α-synuclein. In-gel tryptic digestion of the bands and subsequent LC-MS/MS analysis enabled two unique cross-links to be identified for the NAC–WT Im7 complex (Fig. 7*c* and Table S7). Two unique cross-links were also detected for the NAC–TM Im7 complex (Fig. 7*d* and Table S10). Again, the lack of higher order complexes and the fact that only Lys-79 of Im7 formed detectable cross-links, despite having seven other solvent-accessible lysine residues in each protein, support the specificity of the cross-links observed. Additional cross-links from Lys-79 of TM Im7 to the C-terminal region of β-NAC were also observed. These data therefore suggest that NAC interacts with both folded and unfolded proteins using similar binding regions.

## Discussion

Although many reports have suggested possible functions for NAC (16), much remains to be discovered about its structure and function. First identified in 1994 by Wiedmann *et al.* (47), NAC is now known to be crucial for protein folding and transport in the cell (25, 48). Despite its importance as a chaperone, how NAC binds its client proteins, the role of different domains of NAC in client binding, and the nature of the substrate (whether folded, unfolded or intrinsically disordered) remained unknown. Here, we have used native ESI-MS, limited proteolysis, and cross-linking mapped by MS/MS, and we combined these experiments with CD, NMR, and aggregation assays *in vitro* and *in vivo* to characterize the conformational dynamics of NAC and its ΔUBA variant and to probe the nature of substrate recognition of NAC for three different model protein substrates. Native ESI-MS showed two different charge-state distributions for NAC, suggesting that the αβ-NAC heterodimer is dynamic in structure, visiting compact species as well as more extended states. Although care must be taken in interpreting the conformational properties of dynamic proteins by native MS, especially for unfolded chains and multidomain proteins because collapse of the protein can occur in the gas phase (43), the observation that the termini of NAC are protease-sensitive supports the view that these regions of the protein are dynamic and may unfold to give rise to the more extended species observed by ESI-MS. A similar array of charge states with similar drift times (taking the smaller mass of the complex into consideration) was also observed for the ΔUBA-NAC variant. Thus, the conformational dynamics observed cannot be attributed to the UBA domain, which is known to be connected to the α-NAC domain via a flexible linker (20). This dynamic structure of NAC may be important for imparting its ability to bind a range of protein substrates, including both natively folded and unfolded structures, as shown here using native Im7, unfolded-TM Im7, and α-synuclein as examples.

Collision cross-sections determined using ESI-IMS-MS showed that ΔUBA-NAC has a reduced CCS compared with WT-NAC (Fig. 2*c*). Based on the relationship between the molecular mass and CCS for globular proteins (49, 50), WT-NAC would be expected to have a CCS of ∼2700 Å^2^. The measured value of 2962 Å^2^ for WT-NAC is larger than this value, consistent with NAC containing flexible regions that contribute to the protein being more expanded than globular proteins of similar mass. A CCS of 2761 Å^2^ was measured for ΔUBA-NAC. As ΔUBA-NAC is 4.2 kDa lower in molecular mass than WT-NAC, it would be expected to have a CCS reduced by ∼200 Å^2^. The experimentally determined difference between the lowest charge state of WT-NAC and ΔUBA-NAC was 201 Å^2^, which indicates that the smaller mass is the main factor underlying the reduced CCS and not an altered conformation of the NAC heterodimer upon deletion of the UBA domain.

Although both NAC and ΔUBA-NAC give rise to native ESI mass spectra with similar charge-state distributions, these proteins have different stability in the gas phase as indicated using CIU (Fig. 3). These experiments showed that the absence of the UBA domain results in a complex that is more susceptible to unfolding in the dimeric state. We were unable to detect ubiquitin binding to NAC using native MS (data not shown), and hence the role of the UBA domain in NAC remains unclear. Recently, Ott *et al.* (20) demonstrated that ΔUBA-NAC is a better inhibitor of protein aggregation in *nac*Δ*ssb*Δ yeast cells, suggesting that the ΔUBA-NAC heterodimer could have improved chaperone activity compared with the WT-NAC protein. The fact that ΔUBA-NAC formed an extended conformation at a lower collision energy than the WT-NAC suggests that this variant is more susceptible to undergoing conformational change that may, in part, rationalize the *in vivo* observations of improved chaperone activity.

To determine whether NAC substrates are required to be unfolded such as those that remain unstructured as they emerge from the ribosome exit tunnel (22), or whether NAC can recognize structured domains such as those that fold rapidly upon emergence from the ribosome (51), we used chemical cross-linking coupled with LC-MS/MS to map the binding of NAC to two model proteins (α-synuclein and Im7/TM Im7) as potential substrates. The results showed that NAC binds weakly to the IDP α-synuclein, as well as to Im7 in both its native and unfolded states. A weak interaction was supported by the small but significant NMR chemical shift perturbations specifically involving the acidic C-terminal region of α-synuclein upon NAC binding. The finding that NAC suppresses α-synuclein aggregation both *in vitro* and *in vivo* in *C. elegans* demonstrates that this interaction is functionally relevant (Fig. 5, *b* and *e*). A recent study showed that although ribosome-tethered α-synuclein is a weak substrate for the trigger factor, this chaperone was observed to interact with the first 110 N-terminal residues of the protein (52) Similarly, binding of α-synuclein and Im7/TM Im7 to NAC could not be detected by use of native ESI-MS or native PAGE, consistent with a weak interaction. Such weak binding may be required for ATP-independent chaperones such as NAC, which rely on relatively rapid dissociation to enable substrate folding upon release (45, 52–54). Overall, the results presented demonstrate that NAC can interact with both structured and disordered polypeptides, forming weak and/or transient interactions that predominantly involve the terminal domains of both the α- and β-subunits, at least for the model proteins used here. Whether these binding sites are specific for Im7/TM Im7/α-synuclein or are utilized ubiquitously for other polypeptide chains remains to be seen. Moreover, the results presented indicate that attachment of NAC to the ribosome is not required for binding to the substrates used in this study. Ribosome attachment could alter binding affinity and/or the kinetics of binding, and it may alter the regions of NAC involved in substrate recognition. Indeed, the N-terminal domain of β-NAC may not be involved in substrate binding at the ribosome exit tunnel given that the -RRKKK- motif in this domain is required for ribosome binding (Fig. 1*a*) (21–23). Future work exploiting detailed NMR studies of these complexes, combined with other experiments able to probe weak and transient complexes in residue-specific detail (52), will now be needed to study the interactions of NAC with its clients in atomic detail. Similar studies of the same sequences when nascent on the ribosome (51, 55) will then be able to reveal how the chaperone activity of NAC differs when on and off the ribosome.

## Experimental procedures

### Experimental design and statistical rationale

For CD experiments duplicate samples were analyzed to ensure reproducibility. ESI-IMS-MS, cross-linking, and proteolysis experiments were performed at least in triplicate in separate experiments using freshly prepared samples. Controls included comparing NAC with and without substrate bound so as to reveal differences in conformation upon interaction. Details regarding search parameters and acceptance criteria for MS/MS are given below.

### NAC expression and purification

BL21(DE3) Rosetta cells (Novagen, Merck (UK) Ltd., Watford, UK) were transformed with a plasmid encoding a His_6_-SUMO-NAC construct (25, 48), which contains tandem hexahistidine and SUMO tags at the N terminus of α-NAC. Bacteria were grown overnight on LB-agar plates supplemented with 100 μg/ml ampicillin and 25 μg/ml chloramphenicol. A single colony was used to inoculate a 200-ml culture of LB medium containing 100 μg/ml ampicillin and 25 μg/ml chloramphenicol and incubated overnight at 30 °C with shaking at 120 rpm. The overnight culture was used to inoculate six 1-liter flasks of sterile LB containing 100 μg/ml ampicillin and 25 μg/ml chloramphenicol to an OD_600_ of 0.1. The cultures were incubated at 30 °C and 120 rpm shaking until an OD_600_ of 0.6–0.8 was reached (∼3 h). Protein expression was induced by addition of 1 mm IPTG, and incubation was continued for 5 h after which time cells were harvested at 4400 rpm and 4 °C for 20 min (Beckman Avanti J-26 XP centrifuge, JLA 8.1 rotor; Beckman Coulter (UK) Ltd., High Wycombe, Buckinghamshire, UK). Cells were resuspended in cold Buffer B1 (50 mm sodium phosphate buffer, 300 mm NaCl, 6 mm MgCl_2_, 2 mm DTT, 2 mm PMSF, 10% (v/v) glycerol, pH 8.0) and harvested, and the pellet was stored at −80 °C.

Cells were lysed using a French press (1100 p.s.i.) in Buffer B1 containing protease inhibitors and DNase (20 μg/ml) and then centrifuged at 16,000 rpm at 4 °C for 20 min (Sorvall SS34 rotor). 2.5 g of Protino Ni-IDA matrix (Macherey Nagel GmbH, Duren, Germany) was added to the lysate and incubated with agitation for 1 h at 4 °C. The matrix was then washed with 50 ml of Buffer B1 containing 750 mm NaCl, followed by 50 ml of Buffer B1 where the concentration of NaCl was reduced to 25 mm. NAC was eluted from the matrix using Buffer B1 supplemented with 250 mm imidazole, and fractions containing NAC (as determined by SDS-PAGE) were pooled, and the concentration was assessed using a Bradford assay. Ulp1 (25, 48) was added for SUMO cleavage (8 μg of enzyme per mg of substrate), and NAC was dialyzed into Buffer B2 (20 mm sodium phosphate buffer, 25 mm NaCl, 6 mm MgCl_2_, 2 mm DTT, 5% (v/v) glycerol, pH 7.4). The next day, the NAC/Ulp1 mixture was loaded onto a Resource Q anion-exchange column (6-ml column volume, GE Healthcare) that had been equilibrated with Buffer B2. Proteins were eluted using an increasing gradient of high-salt buffer (Buffer B3: 20 mm sodium phosphate buffer, 650 mm NaCl, 6 mm MgCl_2_, 2 mm DTT, 5% (v/v) glycerol, pH 7.4) over 25 column volumes, and fractions of 1 ml were collected. Fractions containing both cleaved α-NAC (21.8 kDa) and β-NAC (17.5 kDa), as evaluated by SDS-PAGE, were pooled and dialyzed overnight at 4 °C into Buffer B2. Aliquots were frozen in liquid N_2_ and stored at −80 °C.

### α-Synuclein expression and purification

Unlabeled and ^15^N-labeled α-synuclein were expressed recombinantly in *Escherichia coli* BL21 (DE3) cells, and the protein was purified as described previously (56). Cell pellets were resuspended in lysis buffer (25 mm Tris-HCl, pH 8.0, 100 μg/ml lysozyme, 50 μg/ml PMSF, and 20 μg/ml DNase), homogenized, and then heated to 80 °C for 10 min. The homogenate was then centrifuged (35,000 × *g*, 4 °C, 30 min), and the protein, isolated in the soluble fraction, was precipitated twice with 50% (w/v) ammonium sulfate at 4 °C for 30 min. The pellet was resuspended in 20 mm Tris-HCl, pH 8.0, and loaded onto an anion-exchange column (Q-Sepharose, GE Healthcare, Amersham Biosciences, Buckinghamshire, UK), and protein was eluted with a salt gradient. Final salt concentration was 500 mm NaCl in 20 mm Tris-HCl, pH 8. Gel filtration (HiLoadTM 26/60 Superdex 75 preparative grade gel-filtration column using 20 mm sodium phosphate, pH 7.5) was then used as a final purification step. Pure protein was dialyzed against 50 mm ammonium bicarbonate and lyophilized.

### Im7 expression and purification

Im7 constructs were overexpressed in *E. coli* and purified as described previously (40, 41). Briefly, BL21 (DE3) cells transformed with chosen plasmid (containing WT Im7 or TM Im7 (L18A/L19A/L37A)) were cultured in LB medium with carbenicillin (100 μg/ml) selection at 37 °C, 200 rpm. Protein expression was induced using 1 mm IPTG at an OD_600_ = 0.6 and cells grown for a further 5 h. The bacteria were then harvested and lysed using sonication. Im7 proteins were then purified using Ni^2+^-affinity chromatography (Ni^2+^-Sepharose column (5-ml volume) (GE Healthcare, Amersham Biosciences, Buckinghamshire, UK)) using a gradient of 10–500 mm imidazole in 50 mm Tris-HCl, 0.3 m NaCl, pH 8.0. Fractions containing Im7 were pooled, dialyzed into 50 mm sodium phosphate buffer, pH 6.0, and purified further using ion-exchange chromatography (Source15Q resin, 7 ml column (GE Healthcare, Amersham Biosciences, Buckinghamshire, UK)) and gel filtration (300 ml Superdex-75 GL column) in the same buffer. Pure proteins were dialyzed into 18 megohms water and lyophilized.

### Native ESI-IMS-MS

Samples were buffer-exchanged into 100 mm ammonium acetate, pH 6.9, using Micro Bio-Spin^TM^ size-exclusion columns (Bio-Rad, Watford, UK). Native ESI-MS and ESI-IMS-MS experiments were performed on a Synapt HDMS mass spectrometer (Waters UK Ltd., Wilmslow, UK) operating in positive ionization mode with the *m/z* range 500–8000; *m/z* was calibrated with NaI dissolved in 50% (v/v) aqueous 2-propanol. Protein solutions were diluted to 10 μm with ammonium acetate buffer and infused into the mass spectrometer using in-house prepared gold-plated borosilicate capillaries. Typically, an electrospray ionization capillary voltage of 1.4–1.6 kV was applied with a source backing pressure of 4.0 mbar. The cone voltage was set to 40 V, and the source temperature was maintained at 80 °C. Separation in the traveling-wave ion-mobility cell was achieved using a wave velocity of 300 m/s and a ramped wave height from 4 to 12 V with an IMS gas flow (nitrogen) of 25 ml/min. For native MS experiments, the trap collision energy was maintained at 5 V. For collision-induced unfolding (CIU) experiments, the collision voltage was increased in 5-V increments up to 100 V. Data were processed using MassLynx version 4.1 software, and Driftscope version 2.5 was used to extract arrival times from IMS-MS data. Arrival times were calibrated by measuring a series of standard proteins with known collision cross-sections (CCS) under the same experimental conditions as described elsewhere (49). Raw MS data are available at the following DOI: archive.researchdata.leeds.ac.uk/291/.

### Native PAGE

10% native gels were prepared in-house by adding 17 ml of distilled H_2_O to 10 ml of 30% acrylamide (w/v), 0.8% (w/v) bisacrylamide, and 3 ml of 10× Tris/glycine buffer (250 mm Tris-Cl, 1.94 m glycine, pH 8.5). Gels were polymerized by addition of 150 μl of 10% (w/v) ammonium persulfate and 20 μl TEMED. Samples were diluted 2-fold with loading buffer (50 mm Tris-Cl, pH 8.6, 10% (w/v) glycerol, 0.1% (w/v) bromphenol blue) and 20 μl loaded onto the gel alongside NativeMark^TM^ unstained protein standard (Thermo Fisher Scientific). Gels were run at 250 V for 40 min, stained with 0.25% (w/v) Coomassie Blue, 40% (v/v) MeOH, 10% (v/v) acetic acid for 3 h and then destained in 40% (v/v) methanol, 10% (v/v) acetic acid overnight.

### CD spectroscopy (CD)

Far-UV CD spectra were recorded on a Chirascan CD spectrophotometer (Applied Photophysics, Leatherhead, Surrey, UK) using a 1-mm path length cuvette. Proteins were buffer-exchanged into 10 mm sodium phosphate buffer, pH 7.0 and measured at a protein concentration of 10 μm. Three scans were acquired over the range 190–260 nm with a bandwidth of 2.5 nm and a scan speed of 1 nm/s. The three datasets were averaged, and the buffer contribution was subtracted to produce the final spectrum. Secondary structure content was estimated by uploading the data into DichroWeb (57) and using the CONTIN (58) algorithm.

### Limited proteolysis

NAC proteins were buffer-exchanged into 100 mm ammonium acetate, pH 6.9, and diluted to 10 μm before adding trypsin at a 1:500 (w/w) protease/protein ratio (sequencing grade modified trypsin, Promega, UK Ltd., Southampton, UK). Mass spectra were measured after 15 min, 30 min, or 1 h under the same conditions as described above for the native MS experiments. Fragments were assigned manually by comparison with theoretical digest peak lists obtained using the MS-Digest tool in ProteinProspector version 5.20.0 (University of California, San Francisco). The mass tolerance for precursor ions was 20 ppm, and for fragment ions 10 ppm was employed. Raw MS data are available at the following DOI: archive.researchdata.leeds.ac.uk/291/.

### Chemical cross-linking

Substrate proteins were exchanged into 10 mm sodium phosphate buffer, pH 7.0, and added to NAC at a 1:1 molar ratio (20 μm NAC + 20 μm substrate). 1 mg of BS3-*d*_0_ and 1 mg of BS3-*d*_4_ (Thermo Fisher Scientific, Altrincham, Cheshire, UK) were dissolved in 277 μl of sodium phosphate buffer to produce a stock solution of 12.5 mm. The cross-linker was then added to the proteins at 20- or 50-fold molar excess, and the reaction was allowed to proceed at room temperature for 1 h before quenching by the addition of 50 mm Tris-HCl, pH 7.5. Samples were analyzed by Tris-Tricine gels (15% (w/v) acrylamide, 0.4% (w/v) bisacrylamide) followed by staining with InstantBlue^TM^ (Expedeon, San Diego). Gel bands were excised from the gel, cut into 1 × 1-mm pieces, and washed in 500 μl of 25 mm ammonium bicarbonate, pH 7.8, for 1 h with shaking. The solution was removed, and the pieces were destained with 100 μl of 25 mm ammonium bicarbonate in 60% (v/v) acetonitrile. This step was repeated three times. Gel pieces were then dehydrated with 100% acetonitrile (v/v) for 10 min and left to air-dry in a laminar flowhood for 1 h. Rehydration of the gel pieces was achieved by adding 0.1 mg/ml trypsin in 25 mm ammonium bicarbonate and incubating the samples on ice for 30 min. Excess trypsin was then removed, and 25 mm ammonium bicarbonate was added to cover the gel pieces, and the samples were incubated at 37 °C with shaking (1000 rpm) overnight. Peptides were extracted from the gel using three washes with 60% (v/v) acetonitrile, 5% (v/v) formic acid. The extracts were pooled and concentrated using a SpeedVac before being analyzed using a ACQUITY UPLC M-Class coupled to a Synapt HDMS G2Si mass spectrometer (Waters UK Ltd., Wilmslow, UK). Peptides were injected onto a C18 column equilibrated with 0.1% formic acid (v/v) in water and eluted using an increasing gradient of 0.1% (v/v) formic acid in acetonitrile over 60 min at a flow rate of 0. 3 μl/min. The Synapt HDMS G2Si was operated in positive mode using a capillary voltage of 3.0 kV, cone voltage of 40 V, backing pressure of 3.6 mbar, and a trap bias of 2.0 V. The source temperature was 80 °C and the trap pressure was 8.70 × 10^−3^ mbar. Glu-fibrinogen and leucine enkephalin were infused as lock mass calibrants. Data acquisition was achieved using data-dependent analysis with a 1-s MS scan over an *m*/*z* range of 250–3000 and followed by three 1-s MS/MS scans taken from the five most intense ions in the MS spectrum over an *m*/*z* range of 50–2000. Data were acquired using MassLynx version 4.1 and processed using PEAKS Studio 7 (Bioinformatics Solutions, Ontario, Canada). Cross-links were identified using StravroX software (59) and verified manually. Raw MS data are available at the following DOI: archive.researchdata.leeds.ac.uk/291/.

### Analysis of cross-linking data

Raw data files were acquired on an ACQUITY M-Class LC-MS coupled to a Synapt G2Si mass spectrometer. Data files (.raw) were imported into PEAKS Studio version 8.0 for peptide identification from MS/MS data. A false discovery rate of 1% was applied. Data were exported as a Mascot Generic File (mgf) to be imported into StravroX3.6.0. FASTA files of each sequence were imported into StravroX and used to search for theoretical cross-linked peptides that were then compared with the experimental dataset. Threshold score/expectation value for accepting individual spectra was in line with StavroX guidelines. Trypsin was selected as the protease, and cross-linking at Lys, Ser, Thr, and Tyr residues were used as the search parameters. The decoy dataset was used to determine the score threshold above which cross-linked peptides had been assigned with confidence.

### NMR spectroscopy

^1^H-^15^N HSQC spectra were obtained using 50 μm ^15^N-labeled α-synuclein in 10 mm sodium phosphate buffer, pH 7.2, containing 10% (v/v) D_2_O. Spectra were acquired in the absence or presence of equimolar (unlabeled) NAC using a 600-MHz NMR magnet (Oxford Instruments, Plc., Abingdon, UK) with a room temperature probe and an Avance III HD console (Bruker UK Ltd., Coventry, UK). Data were processed and visualized using NMRPipe and CcpNMR analysis software (60). Assignments of ^1^H and HN atoms were transferred from the deposited chemical shifts in the Biological Magnetic Resonance Bank (ID:16543) (38). In cases where transferring assignments was difficult due to resonance overlap, the assignments were confirmed using HNCACB and HNCBCACO triple resonance spectra. Triple resonance spectra were acquired using a sample of 400 μm uniformly ^15^N- and ^13^C-labeled α-synuclein using a Varian Inova spectrometer performing at 600 MHz. Chemical shift differences were calculated using Equation 1,

(1) $$\Delta \delta =\sqrt{{\left(5\;\cdot \;\delta^{1}\;H\right)}^{2}+{\left(\delta^{15}\;N\right)}^{2}}$$

### ThT aggregation kinetics

25-μl samples containing 125 μm α-synuclein in Dulbecco's PBS (Sigma, D8537), 0.02% (w/v) sodium azide, and 10 μm ThT were incubated at 37 °C in sealed 384-well plates (Greiner Bio-One Ltd., UK) in a BMG Clariostar (BMG Labtech Ltd., UK) plate reader using an excitation wavelength of 440 ± 10 nm and emission wavelength of 475 ± 10 nm, with agitation at 600 rpm.

### EM

Transmission electron micrographs were acquired from the ThT aggregation assay samples using a JEM-1400 (JEOL Ltd.) transmission electron microscope. Protein samples were pipetted onto carbon-coated copper grids and stained with 1% (w/v) uranyl acetate solution.

### C. elegans strains and RNAi treatment

*C. elegans* was cultured according to standard techniques (61). Strain NL5901 (pkIs2386 [*unc-54p::*α*-synuclein::YFP* + *unc-119*(+)]) was obtained from the *Caenorhabditis* Genetics Center. RNAi was performed by feeding the worms with *E. coli* HT115(DE3) harboring the vector L4440 to express dsRNA of the respective genes. Simultaneous knockdown of *icd-1* and *icd-2* was achieved as described previously (62).

### Immunoblot analysis and antibodies

Protein samples were applied to BisTris-PAGE and electroblotted onto a nitrocellulose membrane according to standard protocols. Polyclonal antibody against *C. elegans* NAC (1:5000) was described previously (48). As a loading control, anti-actin (1:5000, Santa Cruz Biotechnology) was used.

### Explanation of terms reported in cross-linking data tables

Score indicates the best score calculated for the cross-link within the two peptides. Cross-linked peptides with a score higher than the scores calculated from a decoy dataset (inverted sequences) are more probable. The abbreviations used are as follows: *m/z* indicates mass to charge ratio; *z* indicates peptide charge; M + H^+^ indicates mass of singly charged precursor; calculated indicates theoretical single-charged mass of cross-linked peptide; deviation indicates deviation of theoretical and experimental mass in ppm; peptide 1/2 indicates peptide sequence; protein 1/2 indicates protein from which the peptide is derived; from/to defines the start and stop position of peptide within the protein. Site indicates the residue in the cross-linked peptide that gave the best score.

### Accession codes

The following accession codes were used: α-NAC, >sp|Q86S66-2|1-195; β-NAC, >sp|Q18885|1-161; α-synuclein, >sp|P37840|1-140; Im7, >sp|Q03708|1-87. Im7 contains an additional His-tag (MEH_6_) for purification purposes, which is not observed in the Uniprot database.

## Author contributions

E. M. M., K. G., and T. K. K. formal analysis; E. M. M., M. G., K. G., and T. K. K. investigation; E. M. M. methodology; E. M. M. writing-original draft; E. M. M., M. P. J., M. G., T. K. K., J. R. H., E. D., A. E. A., and S. E. R. writing-review and editing; M. P. J., M. G., J. R. H., and S. E. R. resources; M. P. J. and J. R. H. visualization; M. G., T. K. K., E. D., A. E. A., and S. E. R. conceptualization; E. D. and S. E. R. supervision; E. D., A. E. A., and S. E. R. funding acquisition; E. D., A. E. A., and S. E. R. project administration.

## Supplementary Material

Supporting Information

## Footnotes

6 The abbreviations used are:

NAC: nascent polypeptide–associated complex
BS3: bis-(sulfosuccinimidyl) suberate
ESI-MS: electrospray ionization-mass spectrometry
IDP: intrinsically disordered protein
TM Im7: the triple mutant of the bacterial immunity protein Im7 that contains the amino acid substitutions L18A/L19A/L37A
UBA: ubiquitin-associated domain
ΔUBA-NAC: NAC heterodimer lacking the UBA domain from the α-subunit
IMS-MS: ion-mobility spectrometry–mass spectrometry
CIU: collision induced unfolding
ThT: thioflavin T
CCS: collision cross-section
TEMED: *N*,*N*,*N′*,*N′*-tetramethylethylenediamine
Tricine: *N*-[2-hydroxy-1,1-bis(hydroxymethyl)ethyl]glycine
BisTris: 2-[bis(2-hydroxyethyl)amino]-2-(hydroxymethyl)propane-1,3-diol
PMSF: phenylmethanesulfonyl fluoride
IPTG: isopropyl 1-thio-β-d-galactopyranoside
ER: endoplasmic reticulum
PDB: Protein Data Bank
SUMO: small ubiquitin-like modifier.
