# Supplementary material for: Conformational flexibility within the nascent polypeptide–associated complex enables its interactions with structurally diverse client proteins
Source: J Biol Chem. 2018 Apr 12;293(22):8554–68. doi: 10.1074/jbc.RA117.001568 (PMC5986199; doi:10.1074/jbc.RA117.001568)
Supplement: Supporting Information [file supp_293_22_8554__index.html]

Conformational flexibility within the nascent polypeptide–associated complex enables its interactions with structurally diverse client proteins — Structure and function of NAC — Conformational flexibility within the nascent polypeptide–associated complex enables its interactions with structurally diverse client proteins — Structure and function of NAC — Supporting Information 

# Conformational flexibility within the nascent polypeptide–associated complex enables its interactions with structurally diverse client proteins

## Supporting Information

- Supporting Information - Supporting Information Figures and Tables
- Supporting Information - Supporting Information figures and Tables
