# Supplementary material for: Conformational flexibility within the nascent polypeptide–associated complex enables its interactions with structurally diverse client proteins
Source: J Biol Chem. 2018 Apr 12;293(22):8554–68. doi: 10.1074/jbc.RA117.001568 (PMC5986199; doi:10.1074/jbc.RA117.001568)
Supplement: Supporting Information [file supp_RA117.001568_134820_2_supp_114392_p6tty5.docx]

Supporting information for conformational flexibility within the nascent polypeptide–associated complex enables its interactions with structurally diverse client proteins

**Esther M. Martin^1#,^, Matthew P. Jackson^1^, Martin Gamerdinger^2^, Karina Gense^2^, Theodoros K. Karamonos^1^, Julia R. Humes^1^, Elke Deuerling^2^, Alison E. Ashcroft^1^ and Sheena E. Radford^1*^**

From the ^1^Astbury Centre for Structural Molecular Biology, School of Molecular and Cellular Biology, Faculty of Biological Sciences, University of Leeds, Leeds, LS2 9JT, UK; ^2^Department of Biology, Institute of Molecular Microbiology, University of Konstanz, 78454 Konstanz, Germany

Running title: *Structure and function of NAC*

*^#^*Present address: MedImmune Ltd, Granta Park, Cambridge, CB21 6GH, UK

*To whom correspondence should be addressed: Sheena E. Radford, Astbury Centre for Structural Molecular Biology, School of Molecular and Cellular Biology, Faculty of Biological Sciences, University of Leeds, Leeds, LS2 9JT; [s.e.radford@leeds.ac.uk](mailto:s.e.radford@leeds.ac.uk); +44 113 343 3170.

**Figure S1:** **Native ESI-mass spectrum of WT-NAC after cross-linking with BS3.** WT-NAC was cross-linked with a 50x molar excess of BS3 at room temperature for 30 min. Following this the protein was buffer exchanged into 100 mM ammonium acetate, pH 6.9 and analysed using native ESI-MS. The spectrum shows a loss in intensity of more highly charged ions (>13^+^) generated from more expanded conformers subsequent to chemical cross-linking experiments (compare with Figure 2a).

**Supplementary Tables**

**Table S1: Collisional cross sections of WT NAC and ΔUBA NAC by native mass spectrometry**

| Native conformation (Å^2^) | | | Extended conformation (Å^2^) | | |
| --- | --- | --- | --- | --- | --- |
| Charge state | WT NAC | ΔUBA NAC | **Charge state** | WT NAC | ΔUBA NAC |
| 10 |  | 2693.2 | **16** | 3896.5, 3767.3 | 3741.2 |
| 11 | 2962.3 | 2761.7 | **17** | 4085.7 | 3886.7, 4205.4 |
| 12 | 3048.4 | 2824.5 | **18** | 4239.3 | 4298.1, 4086.2 |
| 13 | 3143.7 | 2925.3 | **19** | 4475.3, 4630.9 | 4537.4 |
| 14 | 3271.6, 3318.9 | 3176.3 | **20** | 4872.7 | 4776.6 |
| 15 | 3767.2, 3628.5 | 3580.5 | **21** | 5083.1 | 4948.5 |
|  |  |  | **22** | 5289.9 | 5109.5 |
|  |  |  | **23** | 5457.9 | 5494.4, 5261.6 |
|  |  |  | **24** | 5950.4, 5732.4 | - |

**Table S2: Estimation of NAC secondary structure by CD spectroscopy.** Values were estimated by importing the data shown in Figure 2d into DichroWeb (1) and using the CONTIN (2).

| Protein | Helix (%) | β-strand (%) | β-turn (%) | Unordered (%) |
| --- | --- | --- | --- | --- |
| WT-NAC | 27.5 | 16.9 | 22.6 | 33.0 |
| ΔUBA-NAC | 15.1 | 27.1 | 21.5 | 36.0 |

**Table S3. Intra- and inter- NAC cross-links observed for the NAC-α-synuclein complex**

| Score | m/z | z | M+H+ | Calculated (Da) | Deviation (Da) | Peptide 1 | Protein 1 | From | To | Peptide 2 | Protein 2 | From | To | Site 1 | Site 2 |
| --- | --- | --- | --- | --- | --- | --- | --- | --- | --- | --- | --- | --- | --- | --- | --- |
| 201 | 711.375 | 3 | 2132.111 | 2132.109 | 0.89 | [VAEAAGLGDHIDKQAKQSR] | alpha-NAC | 39 | 57 | 1 | intrapeptidal | 0 | 0 | K13 | K16 |
| 185 | 594.988 | 3 | 1782.949 | 1782.953 | -2.39 | [QAK] | alpha-NAC | 52 | 54 | [VAEAAGLGDHIDK] | alpha-NAC | 39 | 51 | K3 | K13 |
| 163 | 593.648 | 3 | 1778.93 | 1778.928 | 1.06 | [VAEAAGLGDHIDKQAK] | alpha-NAC | 39 | 54 | 0 | dead-end | 0 | 0 | K16 | x0 |
| 160 | 1140.896 | 3 | 3420.675 | 3420.686 | -3.19 | [DDGTVIHFNNPK] | beta-NAC | 70 | 81 | [VQTSVPANTFSVTGSADNK] | beta-NAC | 82 | 100 | K12 | T3 |
| 99 | 556.283 | 3 | 1666.835 | 1666.832 | 2.16 | [QKEVK] | alpha-NAC | 9 | 13 | {mTGSTETR] | alpha-NAC | 0 | 8 | K2 | {0 |
| 92 | 552.293 | 3 | 1654.864 | 1654.862 | 1.04 | [QKEVK] | alpha-NAC | 9 | 13 | {MTGSTETR] | alpha-NAC | 0 | 8 | K2 | {0 |
| 91 | 418.925 | 3 | 1254.759 | 1254.761 | -0.86 | [SEKK] | alpha-NAC | 58 | 61 | [KLFSK] | alpha-NAC | 64 | 68 | K3 | K1 |
| 86 | 474.312 | 3 | 1420.922 | 1420.919 | 2.35 | [KAR] | alpha-NAC | 61 | 63 | [LFSKLGLK] | alpha-NAC | 65 | 72 | K1 | K4 |
| 86 | 474.312 | 3 | 1420.922 | 1420.919 | 2.35 | [ARK] | alpha-NAC | 62 | 64 | [LFSKLGLK] | alpha-NAC | 65 | 72 | K3 | K4 |
| 79 | 557.624 | 3 | 1670.858 | 1670.857 | 0.7 | [QKEVK] | alpha-NAC | 9 | 13 | {mTGSTETR] | alpha-NAC | 0 | 8 | K2 | {0 |
| 78 | 603.654 | 3 | 1808.948 | 1808.947 | 0.26 | [IGGKGTPR] | beta-NAC | 23 | 30 | {MTGSTETR] | alpha-NAC | 0 | 8 | K4 | {0 |
| 76 | 640.04 | 3 | 1918.106 | 1918.105 | 0.86 | [SKNILFVINKPDVFK] | alpha-NAC | 85 | 99 | 0 | dead-end | 0 | 0 | K2 | x0 |
| 73 | 462.58 | 3 | 1385.725 | 1385.724 | 0.86 | [KSK] | alpha-NAC | 84 | 86 | {MTGSTETR] | alpha-NAC | 0 | 8 | K1 | {0 |
| 68 | 539.314 | 3 | 1615.927 | 1615.928 | -0.81 | [IKKLQAQQEHVR] | beta-NAC | 11 | 22 | 1 | intrapeptidal | 0 | 0 | K2 | K3 |
| 68 | 823.086 | 3 | 2467.242 | 2467.25 | -3.03 | [IEDLTQHAQmSAIENLKPTR] | alpha-NAC | 114 | 133 | 0 | dead-end | 0 | 0 | K17 | x0 |
| 67 | 911.502 | 3 | 2732.491 | 2732.49 | 0.43 | [QITEmLPGILNQLGPESLTHLKK] | beta-NAC | 101 | 123 | 0 | dead-end | 0 | 0 | T19 | x0 |
| 65 | 438.95 | 3 | 1314.837 | 1314.841 | -2.81 | [IKK] | beta-NAC | 11 | 13 | [IGGKGTPR] | beta-NAC | 23 | 30 | K3 | K4 |
| 63 | 733.066 | 3 | 2197.185 | 2197.179 | 2.36 | {mTGSTETR] | alpha-NAC | 0 | 8 | [LGLKQVTGVSR] | alpha-NAC | 69 | 79 | {0 | K4 |
| 60 | 727.733 | 3 | 2181.185 | 2181.185 | 0.16 | {MTGSTETR] | alpha-NAC | 0 | 8 | [LGLKQVTGVSR] | alpha-NAC | 69 | 79 | {0 | K4 |
| 58 | 607.642 | 3 | 1820.911 | 1820.917 | -3.1 | [IGGKGTPR] | beta-NAC | 23 | 30 | {mTGSTETR] | alpha-NAC | 0 | 8 | K4 | {0 |
| 58 | 906.171 | 3 | 2716.498 | 2716.495 | 0.96 | [QITEMLPGILNQLGPESLTHLKK] | beta-NAC | 101 | 123 | 0 | dead-end | 0 | 0 | K23 | x0 |
| 56 | 553.285 | 3 | 1657.841 | 1657.847 | -3.32 | [KLFSK] | alpha-NAC | 64 | 68 | {mTGSTETR] | alpha-NAC | 0 | 8 | K1 | {0 |
| 56 | 911.504 | 3 | 2732.496 | 2732.49 | 2.18 | [QITEmLPGILNQLGPESLTHLKK] | beta-NAC | 101 | 123 | 0 | dead-end | 0 | 0 | T19 | x0 |
| 55 | 731.726 | 3 | 2193.164 | 2193.154 | 4.5 | {mTGSTETR] | alpha-NAC | 0 | 8 | [LGLKQVTGVSR] | alpha-NAC | 69 | 79 | {0 | K4 |
| 55 | 472.969 | 3 | 1416.893 | 1416.894 | -0.63 | [KAR] | alpha-NAC | 61 | 63 | [LFSKLGLK] | alpha-NAC | 65 | 72 | K1 | K8 |
| 55 | 472.969 | 3 | 1416.893 | 1416.894 | -0.63 | [ARK] | alpha-NAC | 62 | 64 | [LFSKLGLK] | alpha-NAC | 65 | 72 | K3 | K8 |
| 55 | 676.734 | 3 | 2028.188 | 2028.189 | -0.68 | [KSKNILFVINKPDVFK] | alpha-NAC | 84 | 99 | 1 | intrapeptidal | 0 | 0 | K1 | K3 |
| 54 | 733.064 | 3 | 2197.178 | 2197.179 | -0.47 | {mTGSTETR] | alpha-NAC | 0 | 8 | [LGLKQVTGVSR] | alpha-NAC | 69 | 79 | {0 | K4 |
| 54 | 823.086 | 3 | 2467.244 | 2467.25 | -2.25 | [IEDLTQHAQmSAIENLKPTR] | alpha-NAC | 114 | 133 | 0 | dead-end | 0 | 0 | K17 | x0 |
| 53 | 437.61 | 3 | 1310.816 | 1310.815 | 0.23 | [IKK] | beta-NAC | 11 | 13 | [IGGKGTPR] | beta-NAC | 23 | 30 | K3 | K4 |
| 52 | 640.041 | 3 | 1918.109 | 1918.105 | 2.11 | [SKNILFVINKPDVFK] | alpha-NAC | 85 | 99 | 0 | dead-end | 0 | 0 | K2 | x0 |
| 48 | 379.914 | 3 | 1137.727 | 1137.729 | -1.81 | [KAR] | alpha-NAC | 61 | 63 | [KLFSK] | alpha-NAC | 64 | 68 | K1 | K1 |
| 48 | 379.914 | 3 | 1137.727 | 1137.729 | -1.81 | [ARK] | alpha-NAC | 62 | 64 | [KLFSK] | alpha-NAC | 64 | 68 | K3 | K1 |
| 48 | 593.647 | 3 | 1778.927 | 1778.928 | -0.99 | [QAK] | alpha-NAC | 52 | 54 | [VAEAAGLGDHIDK] | alpha-NAC | 39 | 51 | K3 | K13 |
| 48 | 593.647 | 3 | 1778.927 | 1778.928 | -0.99 | [VAEAAGLGDHIDKQAK] | alpha-NAC | 39 | 54 | 0 | dead-end | 0 | 0 | K16 | x0 |
| 47 | 602.312 | 3 | 1804.921 | 1804.922 | -0.56 | [IGGKGTPR] | beta-NAC | 23 | 30 | {MTGSTETR] | alpha-NAC | 0 | 8 | K4 | {0 |
| 46 | 643.01 | 3 | 1927.015 | 1927.01 | 2.22 | [KLANNVTK] | beta-NAC | 123 | 130 | {mTGSTETR] | alpha-NAC | 0 | 8 | K1 | {0 |
| 44 | 417.583 | 3 | 1250.736 | 1250.735 | 0.24 | [SEKK] | alpha-NAC | 58 | 61 | [KLFSK] | alpha-NAC | 64 | 68 | K3 | K1 |
| 44 | 712.716 | 3 | 2136.134 | 2136.134 | 0.12 | [LGPDGK] | beta-NAC | 131 | 136 | {MmDSKAIAERIK] | beta-NAC | 0 | 12 | K6 | K12 |
| 44 | 712.716 | 3 | 2136.134 | 2136.134 | 0.12 | [LGPDGK] | beta-NAC | 131 | 136 | {mMDSKAIAERIK] | beta-NAC | 0 | 12 | K6 | K12 |
| 42 | 602.313 | 3 | 1804.925 | 1804.922 | 1.76 | [IGGKGTPR] | beta-NAC | 23 | 30 | {MTGSTETR] | alpha-NAC | 0 | 8 | K4 | T2 |
| 41 | 676.735 | 3 | 2028.191 | 2028.189 | 0.86 | [KSKNILFVINKPDVFK] | alpha-NAC | 84 | 99 | 1 | intrapeptidal | 0 | 0 | K1 | K3 |
| 39 | 711.375 | 3 | 2132.111 | 2132.109 | 1.11 | [LGPDGK] | beta-NAC | 131 | 136 | {MmDSKAIAERIK] | beta-NAC | 0 | 12 | K6 | K5 |
| 39 | 711.375 | 3 | 2132.111 | 2132.109 | 1.11 | [LGPDGK] | beta-NAC | 131 | 136 | {mMDSKAIAERIK] | beta-NAC | 0 | 12 | K6 | K5 |
| 39 | 602.991 | 3 | 1806.957 | 1806.96 | -1.2 | [IGGK] | beta-NAC | 23 | 26 | [VAEAAGLGDHIDK] | alpha-NAC | 39 | 51 | K4 | K13 |
| 39 | 586.296 | 3 | 1756.872 | 1756.88 | -4.34 | [QAKQSR] | alpha-NAC | 52 | 57 | {mTGSTETR] | alpha-NAC | 0 | 8 | K3 | T2 |
| 38 | 538.986 | 3 | 1614.943 | 1614.936 | 3.87 | [LGPDGK] | beta-NAC | 131 | 136 | [KLANNVTK] | beta-NAC | 123 | 130 | K6 | K8 |
| 36 | 604.332 | 3 | 1810.98 | 1810.985 | -2.35 | [IGGK] | beta-NAC | 23 | 26 | [VAEAAGLGDHIDK] | alpha-NAC | 39 | 51 | K4 | K13 |
| 35 | 474.312 | 3 | 1420.922 | 1420.919 | 2.35 | [LGLK] | alpha-NAC | 69 | 72 | [ARKLFSK] | alpha-NAC | 62 | 68 | K4 | K7 |
| 35 | 474.312 | 3 | 1420.922 | 1420.919 | 2.35 | [LGLK] | alpha-NAC | 69 | 72 | [ARKLFSK] | alpha-NAC | 62 | 68 | K4 | K7 |
| 33 | 551.948 | 3 | 1653.83 | 1653.83 | 0.3 | [VCIRK] | alpha-NAC | 80 | 84 | {mTGSTETR] | alpha-NAC | 0 | 8 | K5 | {0 |
| 32 | 731.722 | 3 | 2193.153 | 2193.154 | -0.78 | {mTGSTETR] | alpha-NAC | 0 | 8 | [LGLKQVTGVSR] | alpha-NAC | 69 | 79 | {0 | T7 |
| 31 | 817.757 | 3 | 2451.256 | 2451.255 | 0.48 | [IEDLTQHAQMSAIENLKPTR] | alpha-NAC | 114 | 133 | 0 | dead-end | 0 | 0 | K17 | x0 |
| 29 | 472.969 | 3 | 1416.893 | 1416.894 | -0.63 | [LGLK] | alpha-NAC | 69 | 72 | [ARKLFSK] | alpha-NAC | 62 | 68 | K4 | K7 |
| 29 | 472.969 | 3 | 1416.893 | 1416.894 | -0.63 | [LGLK] | alpha-NAC | 69 | 72 | [ARKLFSK] | alpha-NAC | 62 | 68 | K4 | K7 |
| 29 | 867.145 | 3 | 2599.42 | 2599.413 | 2.74 | [KARKLFSK] | alpha-NAC | 61 | 68 | [SPGSDTYIIFGEAK] | alpha-NAC | 100 | 113 | K1 | T6 |
| 28 | 1139.561 | 3 | 3416.669 | 3416.66 | 2.6 | [DDGTVIHFNNPK] | beta-NAC | 70 | 81 | [VQTSVPANTFSVTGSADNK] | beta-NAC | 82 | 100 | K12 | S4 |
| 28 | 1139.561 | 3 | 3416.669 | 3416.66 | 2.6 | [DDGTVIHFNNPKVQTSVPANTFSVTGSADNK] | beta-NAC | 70 | 100 | 0 | dead-end | 0 | 0 | K12 | x0 |
| 27 | 538.812 | 4 | 2152.225 | 2152.227 | -0.94 | [IKK] | beta-NAC | 11 | 13 | [VAEAAGLGDHIDKQAK] | alpha-NAC | 39 | 54 | K3 | K16 |
| 26 | 712.716 | 3 | 2136.133 | 2136.134 | -0.3 | [LGPDGK] | beta-NAC | 131 | 136 | {MmDSKAIAERIK] | beta-NAC | 0 | 12 | K6 | K5 |
| 26 | 712.716 | 3 | 2136.133 | 2136.134 | -0.3 | [LGPDGK] | beta-NAC | 131 | 136 | {mMDSKAIAERIK] | beta-NAC | 0 | 12 | K6 | K5 |
| 26 | 1032.54 | 3 | 3095.608 | 3095.619 | -3.53 | [DIELVISQANTTR] | alpha-NAC | 160 | 172 | {MTGSTETRQKEVK] | alpha-NAC | 0 | 13 | T12 | K10 |
| 23 | 602.992 | 3 | 1806.961 | 1806.96 | 0.85 | [IGGK] | beta-NAC | 23 | 26 | [VAEAAGLGDHIDK] | alpha-NAC | 39 | 51 | K4 | K13 |
| 158 | 527.739 | 2 | 1054.471 | 1054.472 | -0.83 | {mTGSTETR] | alpha-NAC | 0 | 8 | 0 | dead-end | 0 | 0 | {0 | x0 |
| 133 | 638.102 | 4 | 2549.388 | 2549.398 | -4.16 | [IGGKGTPR] | beta-NAC | 23 | 30 | [VAEAAGLGDHIDKQAK] | alpha-NAC | 39 | 54 | T6 | K16 |
| 131 | 379.232 | 2 | 757.457 | 757.457 | 0.08 | [NKAIR] | alpha-NAC | 173 | 177 | 0 | dead-end | 0 | 0 | K2 | x0 |
| 124 | 851.991 | 2 | 1702.975 | 1702.978 | -1.58 | [NILFVINKPDVFK] | alpha-NAC | 87 | 99 | 0 | dead-end | 0 | 0 | K8 | x0 |
| 118 | 471.275 | 2 | 941.543 | 941.541 | 1.73 | [IGGKGTPR] | beta-NAC | 23 | 30 | 0 | dead-end | 0 | 0 | T6 | x0 |
| 105 | 541.562 | 4 | 2163.224 | 2163.229 | -2.45 | [IGGKGTPR] | beta-NAC | 23 | 30 | [KLQAQQEHVR] | beta-NAC | 13 | 22 | K4 | K1 |
| 103 | 473.287 | 2 | 945.567 | 945.567 | 0.02 | [IGGK] | beta-NAC | 23 | 26 | [GTPR] | beta-NAC | 27 | 30 | K4 | T2 |
| 103 | 741.911 | 2 | 1482.815 | 1482.816 | -1.09 | [LANNVTKLGPDGK] | beta-NAC | 124 | 136 | 0 | dead-end | 0 | 0 | K7 | x0 |
| 102 | 522.308 | 2 | 1043.611 | 1043.609 | 1.06 | [KLANNVTK] | beta-NAC | 123 | 130 | 0 | dead-end | 0 | 0 | K1 | x0 |
| 101 | 471.275 | 2 | 941.543 | 941.541 | 1.73 | [IGGK] | beta-NAC | 23 | 26 | [GTPR] | beta-NAC | 27 | 30 | K4 | T2 |
| 87 | 657.393 | 2 | 1313.779 | 1313.779 | 0.1 | [LGLKQVTGVSR] | alpha-NAC | 69 | 79 | 0 | dead-end | 0 | 0 | K4 | x0 |
| 80 | 493.797 | 2 | 986.587 | 986.588 | -1.06 | [KLQSNLK] | beta-NAC | 46 | 52 | 0 | dead-end | 0 | 0 | K1 | x0 |
| 67 | 851.992 | 2 | 1702.976 | 1702.978 | -1.23 | [NILFVINKPDVFK] | alpha-NAC | 87 | 99 | 0 | dead-end | 0 | 0 | K8 | x0 |
| 60 | 743.923 | 2 | 1486.838 | 1486.841 | -2.37 | [LGPDGK] | beta-NAC | 131 | 136 | [LANNVTK] | beta-NAC | 124 | 130 | K6 | K7 |
| 58 | 569.796 | 4 | 2276.161 | 2276.16 | 0.55 | {mTGSTETR] | alpha-NAC | 0 | 8 | [KLQAQQEHVR] | beta-NAC | 13 | 22 | {0 | K1 |
| 58 | 796.952 | 2 | 1592.896 | 1592.901 | -2.65 | [KLANNVTKLGPDGK] | beta-NAC | 123 | 136 | 1 | intrapeptidal | 0 | 0 | K1 | K8 |
| 57 | 741.911 | 2 | 1482.815 | 1482.816 | -1.09 | [LGPDGK] | beta-NAC | 131 | 136 | [LANNVTK] | beta-NAC | 124 | 130 | K6 | T6 |
| 55 | 696.884 | 2 | 1392.76 | 1392.759 | 0.74 | [KLQAQQEHVR] | beta-NAC | 13 | 22 | 0 | dead-end | 0 | 0 | K1 | x0 |
| 54 | 389.739 | 2 | 778.47 | 778.471 | -1.14 | [KLFSK] | alpha-NAC | 64 | 68 | 0 | dead-end | 0 | 0 | K1 | x0 |
| 53 | 432.646 | 5 | 2159.2 | 2159.204 | -1.91 | [IGGKGTPR] | beta-NAC | 23 | 30 | [KLQAQQEHVR] | beta-NAC | 13 | 22 | K4 | K1 |
| 53 | 659.405 | 2 | 1317.802 | 1317.804 | -1.6 | [LGLK] | alpha-NAC | 69 | 72 | [QVTGVSR] | alpha-NAC | 73 | 79 | K4 | T3 |
| 53 | 659.405 | 2 | 1317.802 | 1317.804 | -1.6 | [LGLK] | alpha-NAC | 69 | 72 | [QVTGVSR] | alpha-NAC | 73 | 79 | K4 | T3 |
| 52 | 657.393 | 2 | 1313.779 | 1313.779 | 0.1 | [LGLK] | alpha-NAC | 69 | 72 | [QVTGVSR] | alpha-NAC | 73 | 79 | K4 | T3 |
| 52 | 657.393 | 2 | 1313.779 | 1313.779 | 0.1 | [LGLK] | alpha-NAC | 69 | 72 | [QVTGVSR] | alpha-NAC | 73 | 79 | K4 | T3 |
| 50 | 891.979 | 2 | 1782.95 | 1782.953 | -1.73 | [QAK] | alpha-NAC | 52 | 54 | [VAEAAGLGDHIDK] | alpha-NAC | 39 | 51 | K3 | K13 |
| 46 | 796.95 | 2 | 1592.893 | 1592.901 | -4.59 | [KLANNVTKLGPDGK] | beta-NAC | 123 | 136 | 1 | intrapeptidal | 0 | 0 | K1 | T7 |
| 43 | 586.377 | 2 | 1171.746 | 1171.745 | 1.26 | [KLFSKLGLK] | alpha-NAC | 64 | 72 | 1 | intrapeptidal | 0 | 0 | K1 | K5 |
| 42 | 661.335 | 4 | 2642.318 | 2642.309 | 3.46 | {MTGSTETR] | alpha-NAC | 0 | 8 | [VAEAAGLGDHIDKQAK] | alpha-NAC | 39 | 54 | T2 | K16 |
| 41 | 820.945 | 2 | 1640.883 | 1640.885 | -1.74 | [TAAADDKKLQSNLK] | beta-NAC | 39 | 52 | 1 | intrapeptidal | 0 | 0 | K7 | K8 |
| 37 | 521.817 | 4 | 2084.244 | 2084.248 | -2.22 | [IGGKGTPR] | beta-NAC | 23 | 30 | [LGLKQVTGVSR] | alpha-NAC | 69 | 79 | K4 | K4 |
| 31 | 854.917 | 4 | 3416.645 | 3416.66 | -4.65 | [DDGTVIHFNNPKVQTSVPANTFSVTGSADNK] | beta-NAC | 70 | 100 | 0 | dead-end | 0 | 0 | K12 | x0 |
| 29 | 854.917 | 4 | 3416.645 | 3416.66 | -4.65 | [DDGTVIHFNNPK] | beta-NAC | 70 | 81 | [VQTSVPANTFSVTGSADNK] | beta-NAC | 82 | 100 | K12 | S4 |
| 29 | 796.984 | 2 | 1592.96 | 1592.956 | 2.69 | [KLFSK] | alpha-NAC | 64 | 68 | [VCIRKSK] | alpha-NAC | 80 | 86 | K1 | K7 |
| 26 | 600.849 | 4 | 2400.375 | 2400.383 | -3.62 | [KAR] | alpha-NAC | 61 | 63 | [KLSVTNIPGIEEVNMIK] | beta-NAC | 53 | 69 | K1 | K1 |
| 26 | 600.849 | 4 | 2400.375 | 2400.383 | -3.62 | [ARK] | alpha-NAC | 62 | 64 | [KLSVTNIPGIEEVNMIK] | beta-NAC | 53 | 69 | K3 | K1 |
| 26 | 855.923 | 4 | 3420.67 | 3420.686 | -4.47 | [DDGTVIHFNNPK] | beta-NAC | 70 | 81 | [VQTSVPANTFSVTGSADNK] | beta-NAC | 82 | 100 | K12 | T3 |
| 25 | 432.646 | 5 | 2159.2 | 2159.204 | -1.91 | [KLQAQQEHVRIGGKGTPR] | beta-NAC | 13 | 30 | 0 | dead-end | 0 | 0 | K1 | x0 |
| 25 | 662.338 | 4 | 2646.331 | 2646.334 | -1.34 | {MTGSTETR] | alpha-NAC | 0 | 8 | [VAEAAGLGDHIDKQAK] | alpha-NAC | 39 | 54 | {0 | K16 |
| 25 | 844.435 | 4 | 3374.717 | 3374.705 | 3.57 | {MmDSK] | beta-NAC | 0 | 5 | {MMDSKAIAERIKKLQAQQEHVR] | beta-NAC | 0 | 22 | {0 | K5 |
| 23 | 600.849 | 4 | 2400.375 | 2400.383 | -3.62 | [KARK] | alpha-NAC | 61 | 64 | [LSVTNIPGIEEVNMIK] | beta-NAC | 54 | 69 | K4 | T4 |
| 22 | 403.487 | 4 | 1610.926 | 1610.934 | -4.73 | [GTPRRK] | beta-NAC | 27 | 32 | [LANNVTK] | beta-NAC | 124 | 130 | K6 | K7 |
| 20 | 855.925 | 4 | 3420.679 | 3420.686 | -1.81 | [DDGTVIHFNNPK] | beta-NAC | 70 | 81 | [VQTSVPANTFSVTGSADNK] | beta-NAC | 82 | 100 | K12 | T9 |
| 19 | 433.452 | 5 | 2163.229 | 2163.229 | 0.01 | [IGGKGTPR] | beta-NAC | 23 | 30 | [KLQAQQEHVR] | beta-NAC | 13 | 22 | K4 | K1 |
| 18 | 404.491 | 4 | 1614.94 | 1614.936 | 2.39 | [LGPDGK] | beta-NAC | 131 | 136 | [KLANNVTK] | beta-NAC | 123 | 130 | K6 | K8 |
| 18 | 662.338 | 4 | 2646.331 | 2646.326 | 1.83 | [LGPDGK] | beta-NAC | 131 | 136 | [VQTSVPANTFSVTGSADNK] | beta-NAC | 82 | 100 | K6 | K19 |

**Table S4. Intra- α-synuclein cross-links observed for the NAC-α-synuclein complex**

| Score | m/z | z | M+H+ | | Calculated (Da) | Deviation (Da) | Peptide 1 | Protein 1 | From | To | Peptide 2 | Protein 2 | From | To | Site 1 | Site 2 |
| --- | --- | --- | --- | --- | --- | --- | --- | --- | --- | --- | --- | --- | --- | --- | --- | --- |
| 144 | 668.872 | 2 | | 1336.736 | 1336.736 | 0.05 | [EGVLYVGSKTK] | alpha-synuclein | 35 | 45 | 0 | dead-end | 0 | 0 | T10 | x0 |
| 125 | 865.807 | 3 | | 2595.407 | 2595.403 | 1.62 | [EGVLYVGSKTKEGVVHGVATVAEK] | alpha-synuclein | 35 | 58 | 1 | intrapeptidal | 0 | 0 | K9 | T10 |
| 85 | 505.611 | 3 | | 1514.82 | 1514.826 | -4.2 | [GLSKAK] | alpha-synuclein | 7 | 12 | {MDVFMK] | alpha-synuclein | 0 | 6 | K6 | {0 |
| 82 | 771.758 | 3 | | 2313.26 | 2313.266 | -2.45 | [TKEQVTNVGGAVVTGVTAVAQK] | alpha-synuclein | 59 | 80 | 0 | dead-end | 0 | 0 | K2 | x0 |
| 80 | 893.736 | 4 | | 3571.922 | 3571.933 | -3.08 | [EGVVHGVATVAEKTKEQVTNVGGAVVTGVTAVAQK] | alpha-synuclein | 46 | 80 | 1 | intrapeptidal | 0 | 0 | K15 | T14 |
| 79 | 608.333 | 2 | | 1215.658 | 1215.658 | 0.31 | [TKQGVAEAAGK] | alpha-synuclein | 22 | 32 | 0 | dead-end | 0 | 0 | K2 | x0 |
| 74 | 798.454 | 2 | | 1595.901 | 1595.9 | 0.39 | [GLSKAKEGVVAAAEK] | alpha-synuclein | 7 | 21 | 1 | intrapeptidal | 0 | 0 | K4 | K6 |
| 64 | 560.978 | 3 | | 1680.918 | 1680.917 | 0.83 | [TKEGVVHGVATVAEK] | alpha-synuclein | 44 | 58 | 0 | dead-end | 0 | 0 | K2 | x0 |
| 64 | 684.703 | 3 | | 2052.094 | 2052.097 | -1.32 | [EGVVAAAEKTKQGVAEAAGK] | alpha-synuclein | 13 | 32 | 1 | intrapeptidal | 0 | 0 | K11 | T10 |
| 63 | 672.684 | 3 | | 2016.036 | 2016.033 | 1.47 | {mDVFmK] | alpha-synuclein | 0 | 6 | [AKEGVVAAAEK] | alpha-synuclein | 11 | 21 | {0 | K2 |
| 59 | 422.741 | 2 | | 844.476 | 844.477 | -2.15 | [KDQLGK] | alpha-synuclein | 97 | 102 | 0 | dead-end | 0 | 0 | K1 | x0 |
| 57 | 614.842 | 2 | | 1228.677 | 1228.678 | -1.48 | [AKEGVVAAAEK] | alpha-synuclein | 11 | 21 | 0 | dead-end | 0 | 0 | K2 | x0 |
| 55 | 569.07 | 4 | | 2273.26 | 2273.265 | -2.25 | [TKQGVAEAAGK] | alpha-synuclein | 22 | 32 | [AKEGVVAAAEK] | alpha-synuclein | 11 | 21 | K2 | K2 |
| 51 | 881.98 | 2 | | 1762.952 | 1762.959 | -3.59 | [TVEGAGSIAAATGFVKK] | alpha-synuclein | 81 | 97 | 0 | dead-end | 0 | 0 | T12 | x0 |
| 50 | 560.977 | 3 | | 1680.918 | 1680.917 | 0.51 | [TKEGVVHGVATVAEK] | alpha-synuclein | 44 | 58 | 0 | dead-end | 0 | 0 | T1 | x0 |
| 49 | 672.683 | 3 | | 2016.035 | 2016.033 | 1.17 | {mDVFmK] | alpha-synuclein | 0 | 6 | [AKEGVVAAAEK] | alpha-synuclein | 11 | 21 | {0 | K2 |
| 48 | 840.96 | 2 | | 1680.913 | 1680.917 | -2.23 | [TKEGVVHGVATVAEK] | alpha-synuclein | 44 | 58 | 0 | dead-end | 0 | 0 | T1 | x0 |
| 48 | 1026.55 | 2 | | 2052.094 | 2052.097 | -1.19 | [EGVVAAAEKTKQGVAEAAGK] | alpha-synuclein | 13 | 32 | 1 | intrapeptidal | 0 | 0 | K9 | K11 |
| 46 | 655.33 | 2 | | 1309.652 | 1309.653 | -0.71 | {mDVFMKGLSK] | alpha-synuclein | 0 | 10 | 1 | intrapeptidal | 0 | 0 | {0 | K6 |
| 46 | 655.33 | 2 | | 1309.652 | 1309.653 | -0.71 | {MDVFmKGLSK] | alpha-synuclein | 0 | 10 | 1 | intrapeptidal | 0 | 0 | {0 | K6 |
| 45 | 712.403 | 4 | | 2846.591 | 2846.581 | 3.31 | [TKEGVLYVGSK] | alpha-synuclein | 33 | 43 | [TKEGVVHGVATVAEK] | alpha-synuclein | 44 | 58 | T1 | K2 |
| 45 | 663.329 | 2 | | 1325.651 | 1325.648 | 2.6 | {mDVFmKGLSK] | alpha-synuclein | 0 | 10 | 1 | intrapeptidal | 0 | 0 | {0 | K6 |
| 45 | 671.342 | 3 | | 2012.011 | 2012.008 | 1.48 | {mDVFmK] | alpha-synuclein | 0 | 6 | [AKEGVVAAAEK] | alpha-synuclein | 11 | 21 | {0 | K2 |
| 41 | 569.07 | 4 | | 2273.26 | 2273.265 | -2.25 | [QGVAEAAGKTK] | alpha-synuclein | 24 | 34 | [AKEGVVAAAEK] | alpha-synuclein | 11 | 21 | K11 | K2 |
| 39 | 712.403 | 4 | | 2846.591 | 2846.581 | 3.31 | [TKEGVLYVGSK] | alpha-synuclein | 33 | 43 | [EGVVHGVATVAEKTK] | alpha-synuclein | 46 | 60 | T1 | K15 |
| 38 | 568.065 | 4 | | 2269.237 | 2269.24 | -1.13 | [TKQGVAEAAGK] | alpha-synuclein | 22 | 32 | [AKEGVVAAAEK] | alpha-synuclein | 11 | 21 | K2 | K2 |
| 37 | 629.849 | 2 | | 1258.691 | 1258.689 | 1.76 | [EGVVAAAEKTK] | alpha-synuclein | 13 | 23 | 0 | dead-end | 0 | 0 | K11 | x0 |
| 35 | 671.338 | 3 | | 2011.999 | 2012.008 | -4.34 | {mDVFmK] | alpha-synuclein | 0 | 6 | [AKEGVVAAAEK] | alpha-synuclein | 11 | 21 | K6 | K2 |
| 35 | 516.275 | 3 | | 1546.814 | 1546.816 | -1.35 | [GLSKAK] | alpha-synuclein | 7 | 12 | {mDVFmK] | alpha-synuclein | 0 | 6 | K6 | {0 |
| 35 | 532.637 | 3 | | 1595.897 | 1595.9 | -2.04 | [GLSKAKEGVVAAAEK] | alpha-synuclein | 7 | 21 | 1 | intrapeptidal | 0 | 0 | K4 | K6 |
| 34 | 616.366 | 3 | | 1847.082 | 1847.079 | 2.06 | [GLSKAK] | alpha-synuclein | 7 | 12 | [EGVVAAAEKTK] | alpha-synuclein | 13 | 23 | K6 | K11 |
| 32 | 514.933 | 3 | | 1542.786 | 1542.791 | -3.22 | [GLSKAK] | alpha-synuclein | 7 | 12 | {mDVFmK] | alpha-synuclein | 0 | 6 | K6 | {0 |
| 30 | 720.409 | 2 | | 1439.811 | 1439.81 | 0.28 | [AKEGVVAAAEKTK] | alpha-synuclein | 11 | 23 | 1 | intrapeptidal | 0 | 0 | K2 | T12 |
| 30 | 878.978 | 2 | | 1756.948 | 1756.944 | 2.33 | [DQLGK] | alpha-synuclein | 98 | 102 | [QGVAEAAGKTK] | alpha-synuclein | 24 | 34 | K5 | K11 |
| 30 | 758.426 | 3 | | 2273.262 | 2273.265 | -1.25 | [TKQGVAEAAGK] | alpha-synuclein | 22 | 32 | [AKEGVVAAAEK] | alpha-synuclein | 11 | 21 | T1 | K11 |
| 30 | 757.084 | 3 | | 2269.238 | 2269.233 | 2.37 | [EGVLYVGSK] | alpha-synuclein | 35 | 43 | [TKEGVLYVGSK] | alpha-synuclein | 33 | 43 | Y5 | K11 |
| 30 | 514.933 | 3 | | 1542.785 | 1542.791 | -3.41 | [GLSKAK] | alpha-synuclein | 7 | 12 | {mDVFmK] | alpha-synuclein | 0 | 6 | K4 | {0 |
| 30 | 1032.214 | 3 | | 3094.627 | 3094.631 | -1.04 | [TVEGAGSIAAATGFVK] | alpha-synuclein | 81 | 96 | [TVEGAGSIAAATGFVK] | alpha-synuclein | 81 | 96 | K16 | S7 |
| 28 | 568.065 | 4 | | 2269.237 | 2269.24 | -1.13 | [QGVAEAAGKTK] | alpha-synuclein | 24 | 34 | [AKEGVVAAAEK] | alpha-synuclein | 11 | 21 | K11 | K2 |
| 23 | 538.812 | 4 | | 2152.225 | 2152.216 | 4.28 | [GLSK] | alpha-synuclein | 7 | 10 | [TVEGAGSIAAATGFVKK] | alpha-synuclein | 81 | 97 | K4 | K17 |
| 22 | 595.079 | 4 | | 2377.296 | 2377.297 | -0.73 | [TKQGVAEAAGK] | alpha-synuclein | 22 | 32 | [TKEGVLYVGSK] | alpha-synuclein | 33 | 43 | K2 | K2 |
| 19 | 595.079 | 4 | | 2377.296 | 2377.297 | -0.73 | [TKQGVAEAAGK] | alpha-synuclein | 22 | 32 | [EGVLYVGSKTK] | alpha-synuclein | 35 | 45 | K2 | K11 |

**Table S5. NAC-α-synuclein cross-links observed for the NAC-α-synuclein complex**

| Score | m/z | z | M+H+ | Calculated (Da) | Deviation (Da) | Peptide 1 | Protein 1 | From | To | Peptide 2 | Protein 2 | From | To | Site 1 | Site 2 |
| --- | --- | --- | --- | --- | --- | --- | --- | --- | --- | --- | --- | --- | --- | --- | --- |
| 53 | 865.807 | 3 | 2595.407 | 2595.418 | -4.13 | [EGVLYVGSK] | alpha-synuclein | 35 | 43 | [TAAADDKKLQSNLK] | beta-NAC | 39 | 52 | Y5 | K8 |
| 51 | 404.491 | 4 | 1614.94 | 1614.948 | -4.55 | [KDQLGK] | alpha-synuclein | 97 | 102 | [IGGKGTPR] | beta-NAC | 23 | 30 | K1 | K4 |
| 44 | 1037.54 | 3 | 3110.607 | 3110.604 | 1.01 | [TVEGAGSIAAATGFVK] | alpha-synuclein | 81 | 96 | {MTGSTETRQKEVK] | alpha-NAC | 0 | 13 | S7 | K10 |
| 38 | 722.42 | 2 | 1443.833 | 1443.835 | -1.85 | [VCIRK] | alpha-NAC | 80 | 84 | [KDQLGK] | alpha-synuclein | 97 | 102 | K5 | K1 |
| 36 | 586.377 | 2 | 1171.746 | 1171.752 | -4.88 | [RKK] | beta-NAC | 31 | 33 | [GLSKAK] | alpha-synuclein | 7 | 12 | K2 | K4 |
| 35 | 516.304 | 3 | 1546.897 | 1546.895 | 0.9 | [DQLGK] | alpha-synuclein | 98 | 102 | [ARKLFSK] | alpha-NAC | 62 | 68 | K5 | S6 |
| 33 | 594.99 | 3 | 1782.954 | 1782.96 | -3 | [LGPDGK] | beta-NAC | 131 | 136 | [QGVAEAAGKTK] | alpha-synuclein | 24 | 34 | K6 | K11 |
| 33 | 594.99 | 3 | 1782.954 | 1782.96 | -3 | [LGPDGK] | beta-NAC | 131 | 136 | [TKQGVAEAAGK] | alpha-synuclein | 22 | 32 | K6 | K11 |
| 33 | 634.114 | 4 | 2533.433 | 2533.429 | 1.92 | [IGGKGTPR] | beta-NAC | 23 | 30 | [TVEGAGSIAAATGFVKK] | alpha-synuclein | 81 | 97 | K4 | K17 |
| 32 | 712.716 | 3 | 2136.134 | 2136.13 | 2.25 | [QGVAEAAGK] | alpha-synuclein | 24 | 32 | [VIHKTAAADDK] | beta-NAC | 35 | 45 | K9 | K11 |
| 32 | 798.134 | 4 | 3189.515 | 3189.516 | -0.07 | {MDVFMKGLSK] | alpha-synuclein | 0 | 10 | [GEDEDVPELVGDFDAASK] | beta-NAC | 137 | 154 | K6 | K18 |
| 28 | 538.986 | 3 | 1614.943 | 1614.948 | -3.09 | [KDQLGK] | alpha-synuclein | 97 | 102 | [IGGKGTPR] | beta-NAC | 23 | 30 | K6 | T6 |
| 26 | 741.911 | 2 | 1482.815 | 1482.817 | -1.67 | [RKKK] | beta-NAC | 31 | 34 | {MDVFmK] | alpha-synuclein | 0 | 6 | K3 | {0 |
| 18 | 1063.95 | 5 | 5315.719 | 5315.697 | 4.25 | [EGVVAAAEKTKQGVAEAAGK] | alpha-synuclein | 13 | 32 | [DDGTVIHFNNPKVQTSVPANTFSVTGSADNK] | beta-NAC | 70 | 100 | K20 | K31 |

**Table S6. Intra- and inter- NAC cross-links observed for the NAC-WT Im7 complex**

| Score | m/z | z | M+H+ | Calculated (Da) | Deviation (Da) | Peptide 1 | Protein 1 | From | To | Peptide 2 | Protein 2 | From | To | Site 1 | Site 2 |
| --- | --- | --- | --- | --- | --- | --- | --- | --- | --- | --- | --- | --- | --- | --- | --- |
| 120 | 550.951 | 3 | 1650.838 | 1650.837 | 0.82 | [QKEVK] | alpha-NAC | 9 | 13 | {MTGSTETR] | alpha-NAC | 0 | 8 | K2 | {0 |
| 105 | 607.643 | 3 | 1820.916 | 1820.917 | -0.79 | [IGGKGTPR] | beta-NAC | 23 | 30 | {mTGSTETR] | alpha-NAC | 0 | 8 | K4 | {0 |
| 101 | 636.337 | 3 | 1906.996 | 1906.99 | 3.09 | {MTGSTETR] | alpha-NAC | 0 | 8 | [KLANNVTK] | beta-NAC | 123 | 130 | {0 | T7 |
| 85 | 602.311 | 3 | 1804.918 | 1804.922 | -2.56 | [IGGKGTPR] | beta-NAC | 23 | 30 | {MTGSTETR] | alpha-NAC | 0 | 8 | K4 | {0 |
| 77 | 556.281 | 3 | 1666.83 | 1666.832 | -1.19 | [QKEVK] | alpha-NAC | 9 | 13 | {mTGSTETR] | alpha-NAC | 0 | 8 | K2 | {0 |
| 76 | 641.667 | 3 | 1922.987 | 1922.985 | 0.74 | [KLANNVTK] | beta-NAC | 123 | 130 | {mTGSTETR] | alpha-NAC | 0 | 8 | K1 | {0 |
| 70 | 618.962 | 3 | 1854.873 | 1854.875 | -1.13 | [TAAADDKK] | beta-NAC | 39 | 46 | {mTGSTETR] | alpha-NAC | 0 | 8 | K7 | {0 |
| 69 | 731.725 | 3 | 2193.161 | 2193.154 | 3 | {mTGSTETR] | alpha-NAC | 0 | 8 | [LGLKQVTGVSR] | alpha-NAC | 69 | 79 | {0 | S4 |
| 63 | 607.643 | 3 | 1820.914 | 1820.917 | -1.45 | [IGGKGTPR] | beta-NAC | 23 | 30 | {mTGSTETR] | alpha-NAC | 0 | 8 | K4 | {0 |
| 60 | 613.633 | 3 | 1838.886 | 1838.88 | 2.99 | [TAAADDKK] | beta-NAC | 39 | 46 | {MTGSTETR] | alpha-NAC | 0 | 8 | K7 | {0 |
| 60 | 970.828 | 3 | 2910.471 | 2910.485 | -4.97 | {mTGSTETR] | alpha-NAC | 0 | 8 | [LQAQQEHVRIGGKGTPR] | beta-NAC | 14 | 30 | T2 | K13 |
| 60 | 584.955 | 3 | 1752.849 | 1752.854 | -2.9 | [QAKQSR] | alpha-NAC | 52 | 57 | {mTGSTETR] | alpha-NAC | 0 | 8 | K3 | T2 |
| 53 | 602.311 | 3 | 1804.918 | 1804.922 | -2.56 | [IGGKGTPR] | beta-NAC | 23 | 30 | {MTGSTETR] | alpha-NAC | 0 | 8 | K4 | T2 |
| 49 | 613.632 | 3 | 1838.88 | 1838.88 | 0.2 | [TAAADDKK] | beta-NAC | 39 | 46 | {MTGSTETR] | alpha-NAC | 0 | 8 | K7 | {0 |
| 42 | 881.444 | 3 | 2642.317 | 2642.309 | 3.02 | {MTGSTETR] | alpha-NAC | 0 | 8 | [VAEAAGLGDHIDKQAK] | alpha-NAC | 39 | 54 | T2 | K13 |
| 131 | 531.292 | 4 | 2122.146 | 2122.15 | -2.06 | [KSK] | alpha-NAC | 84 | 86 | [VAEAAGLGDHIDKQAK] | alpha-NAC | 39 | 54 | K3 | K16 |
| 124 | 540.554 | 4 | 2159.194 | 2159.204 | -4.9 | [IGGKGTPR] | beta-NAC | 23 | 30 | [KLQAQQEHVR] | beta-NAC | 13 | 22 | K4 | K1 |
| 123 | 537.804 | 4 | 2148.195 | 2148.202 | -3.52 | [IKK] | beta-NAC | 11 | 13 | [VAEAAGLGDHIDKQAK] | alpha-NAC | 39 | 54 | K3 | K16 |
| 115 | 471.272 | 2 | 941.537 | 941.541 | -4.38 | [IGGK] | beta-NAC | 23 | 26 | [GTPR] | beta-NAC | 27 | 30 | K4 | T2 |
| 81 | 428.926 | 3 | 1284.762 | 1284.763 | -0.98 | [KSK] | alpha-NAC | 84 | 86 | [IGGKGTPR] | beta-NAC | 23 | 30 | K3 | K4 |
| 77 | 741.911 | 2 | 1482.815 | 1482.816 | -0.75 | [LGPDGK] | beta-NAC | 131 | 136 | [LANNVTK] | beta-NAC | 124 | 130 | K6 | K6 |
| 66 | 759.768 | 5 | 3794.812 | 3794.824 | -3.27 | [ARKLFSK] | alpha-NAC | 62 | 68 | [GEDEDVPELVGDFDAASKNETKADEQ} | beta-NAC | 137 | 163 | S6 | K18 |
| 60 | 602.992 | 3 | 1806.96 | 1806.96 | 0.36 | [IGGK] | beta-NAC | 23 | 26 | [VAEAAGLGDHIDK] | alpha-NAC | 39 | 51 | K4 | K13 |
| 59 | 657.393 | 2 | 1313.778 | 1313.779 | -0.46 | [LGLK] | alpha-NAC | 69 | 72 | [QVTGVSR] | alpha-NAC | 73 | 79 | K4 | T3 |
| 56 | 471.274 | 2 | 941.54 | 941.541 | -1.42 | [IGGK] | beta-NAC | 23 | 26 | [GTPR] | beta-NAC | 27 | 30 | K4 | T2 |
| 56 | 854.918 | 4 | 3416.648 | 3416.66 | -3.55 | [DDGTVIHFNNPK] | beta-NAC | 70 | 81 | [VQTSVPANTFSVTGSADNK] | beta-NAC | 82 | 100 | K12 | S4 |
| 52 | 432.646 | 5 | 2159.199 | 2159.204 | -2.6 | [IGGKGTPR] | beta-NAC | 23 | 30 | [KLQAQQEHVR] | beta-NAC | 13 | 22 | K4 | K1 |
| 49 | 889.969 | 2 | 1778.931 | 1778.928 | 1.47 | [QAK] | alpha-NAC | 52 | 54 | [VAEAAGLGDHIDK] | alpha-NAC | 39 | 51 | K3 | K13 |
| 47 | 537.804 | 4 | 2148.195 | 2148.202 | -3.52 | [LGPDGK] | beta-NAC | 131 | 136 | [KKVIHKTAAADDK] | beta-NAC | 33 | 45 | K6 | K13 |
| 47 | 639.869 | 4 | 2556.454 | 2556.462 | -3.07 | [LGPDGK] | beta-NAC | 131 | 136 | [IKKLQAQQEHVRIGGK] | beta-NAC | 11 | 26 | K6 | K3 |
| 46 | 889.968 | 2 | 1778.928 | 1778.928 | -0.16 | [QAK] | alpha-NAC | 52 | 54 | [VAEAAGLGDHIDK] | alpha-NAC | 39 | 51 | K3 | K13 |
| 39 | 428.926 | 3 | 1284.762 | 1284.763 | -0.98 | [LGLK] | alpha-NAC | 69 | 72 | [QAKQSR] | alpha-NAC | 52 | 57 | K4 | S5 |
| 37 | 463.777 | 4 | 1852.087 | 1852.09 | -1.58 | [IKK] | beta-NAC | 11 | 13 | [LANNVTKLGPDGK] | beta-NAC | 124 | 136 | K3 | K7 |
| 37 | 854.92 | 4 | 3416.659 | 3416.66 | -0.47 | [DDGTVIHFNNPK] | beta-NAC | 70 | 81 | [VQTSVPANTFSVTGSADNK] | beta-NAC | 82 | 100 | K12 | T3 |
| 34 | 871.472 | 2 | 1741.937 | 1741.944 | -4.34 | [IGGKGTPR] | beta-NAC | 23 | 30 | [TAAADDKK] | beta-NAC | 39 | 46 | K4 | K7 |
| 33 | 687.867 | 2 | 1374.728 | 1374.722 | 3.78 | [NETK] | beta-NAC | 155 | 158 | [QVTGVSR] | alpha-NAC | 73 | 79 | K4 | S6 |
| 32 | 530.311 | 2 | 1059.615 | 1059.616 | -1.09 | [RKK] | beta-NAC | 31 | 33 | [NETK] | beta-NAC | 155 | 158 | K2 | K4 |
| 31 | 534.293 | 4 | 2134.151 | 2134.15 | 0.15 | [IGGK] | beta-NAC | 23 | 26 | [VAEAAGLGDHIDKQAK] | alpha-NAC | 39 | 54 | K4 | K16 |
| 30 | 430.445 | 5 | 2148.197 | 2148.202 | -2.61 | [IKK] | beta-NAC | 11 | 13 | [VAEAAGLGDHIDKQAK] | alpha-NAC | 39 | 54 | K3 | K13 |
| 30 | 531.333 | 2 | 1061.658 | 1061.661 | -2.62 | [LGLK] | alpha-NAC | 69 | 72 | [LFSK] | alpha-NAC | 65 | 68 | K4 | K4 |
| 25 | 512.1 | 5 | 2556.471 | 2556.462 | 3.66 | [LGPDGK] | beta-NAC | 131 | 136 | [IKKLQAQQEHVRIGGK] | beta-NAC | 11 | 26 | K6 | K16 |
| 24 | 395.981 | 4 | 1580.903 | 1580.901 | 1.4 | [TAAADDK] | beta-NAC | 39 | 45 | [KKVIHK] | beta-NAC | 33 | 38 | K7 | K6 |
| 24 | 395.981 | 4 | 1580.903 | 1580.901 | 1.4 | [KVIHK] | beta-NAC | 34 | 38 | [TAAADDKK] | beta-NAC | 39 | 46 | K5 | K8 |
| 24 | 537.805 | 4 | 2148.199 | 2148.202 | -1.73 | [IKK] | beta-NAC | 11 | 13 | [VAEAAGLGDHIDKQAK] | alpha-NAC | 39 | 54 | K3 | K16 |

**Table S7. NAC-WT Im7 cross-links observed for the NAC-WT Im7 complex**

| Score | m/z | z | M+H+ | Calculated (Da) | Deviation (Da) | Peptide 1 | Protein 1 | From | To | Peptide 2 | Protein 2 | From | To | Site 1 | Site 2 |
| --- | --- | --- | --- | --- | --- | --- | --- | --- | --- | --- | --- | --- | --- | --- | --- |
| 56 | 627.31 | 3 | 1879.92 | 1879.92 | -3.24 | [EIKEWR] | WT Im7 | 77 | 82 | {MTGSTETR] | alpha-NAC | 0 | 8 | K3 | {0 |
| 98 | 789.055 | 3 | 2365.15 | 2365.14 | 3.02 | {mTGSTETR] | alpha-NAC | 0 | 8 | [DDSPEGIVKEIK] | WT Im7 | 68 | 79 | S4 | K9 |

**Table S8. Intra- and inter- NAC cross-links observed for the NAC-TM-Im7 complex**

| Score | m/z | z | M+H+ | Calculated (Da) | Deviation (Da) | Peptide 1 | Protein 1 | From | To | Peptide 2 | Protein 2 | From | To | Site 1 | Site 2 |
| --- | --- | --- | --- | --- | --- | --- | --- | --- | --- | --- | --- | --- | --- | --- | --- |
| 231 | 593.647 | 3 | 1778.927 | 1778.928 | -0.77 | [QAK] | alpha-NAC | 52 | 54 | [VAEAAGLGDHIDK] | alpha-NAC | 39 | 51 | K3 | K13 |
| 113 | 1139.556 | 3 | 3416.654 | 3416.66 | -1.79 | [DDGTVIHFNNPK] | beta-NAC | 70 | 81 | [VQTSVPANTFSVTGSADNK] | beta-NAC | 82 | 100 | K12 | T3 |
| 96 | 504.252 | 3 | 1510.743 | 1510.742 | 0.67 | [SEKK] | alpha-NAC | 58 | 61 | {MTGSTETR] | alpha-NAC | 0 | 8 | K3 | {0 |
| 93 | 550.949 | 3 | 1650.832 | 1650.837 | -2.76 | [QKEVK] | alpha-NAC | 9 | 13 | {MTGSTETR] | alpha-NAC | 0 | 8 | K2 | {0 |
| 68 | 602.311 | 3 | 1804.917 | 1804.922 | -2.73 | [IGGKGTPR] | beta-NAC | 23 | 30 | {MTGSTETR] | alpha-NAC | 0 | 8 | K4 | {0 |
| 65 | 437.609 | 3 | 1310.813 | 1310.815 | -1.6 | [IKK] | beta-NAC | 11 | 13 | [IGGKGTPR] | beta-NAC | 23 | 30 | K3 | K4 |
| 60 | 461.239 | 3 | 1381.701 | 1381.699 | 1.51 | [KSK] | alpha-NAC | 84 | 86 | {MTGSTETR] | alpha-NAC | 0 | 8 | K1 | {0 |
| 55 | 602.313 | 3 | 1804.925 | 1804.922 | 1.43 | [IGGKGTPR] | beta-NAC | 23 | 30 | {MTGSTETR] | alpha-NAC | 0 | 8 | K4 | {0 |
| 52 | 556.279 | 3 | 1666.823 | 1666.832 | -4.97 | [QKEVK] | alpha-NAC | 9 | 13 | {mTGSTETR] | alpha-NAC | 0 | 8 | K2 | {0 |
| 52 | 726.393 | 3 | 2177.163 | 2177.159 | 1.8 | {MTGSTETR] | alpha-NAC | 0 | 8 | [LGLKQVTGVSR] | alpha-NAC | 69 | 79 | {0 | K4 |
| 45 | 607.645 | 3 | 1820.921 | 1820.917 | 2 | [IGGKGTPR] | beta-NAC | 23 | 30 | {mTGSTETR] | alpha-NAC | 0 | 8 | K4 | {0 |
| 44 | 711.374 | 3 | 2132.107 | 2132.109 | -1 | [LGPDGK] | beta-NAC | 131 | 136 | {mMDSKAIAERIK] | beta-NAC | 0 | 12 | K6 | K12 |
| 37 | 579.626 | 3 | 1736.862 | 1736.86 | 1.54 | [QAKQSR] | alpha-NAC | 52 | 57 | {MTGSTETR] | alpha-NAC | 0 | 8 | K3 | {0 |
| 33 | 628.005 | 3 | 1882.001 | 1881.992 | 4.8 | [IGGKGTPR] | beta-NAC | 23 | 30 | [DDSPEGIVK] | TM Im7 | 68 | 76 | K4 | K9 |
| 30 | 879.434 | 3 | 2636.288 | 2636.291 | -0.85 | [QSRSEKK] | alpha-NAC | 55 | 61 | [EADNDIVNAIMSLTM} | alpha-NAC | 181 | 196 | K6 | T14 |
| 29 | 803.757 | 3 | 2409.257 | 2409.266 | -3.89 | {mmDSK] | beta-NAC | 0 | 5 | [LGLKQVTGVSRVCIR] | alpha-NAC | 69 | 83 | {0 | T7 |
| 28 | 1032.872 | 3 | 3096.602 | 3096.599 | 1.08 | [EVK] | alpha-NAC | 11 | 13 | [EIEKENVAATDDVLDVALEHFVK] | TM Im7 | 27 | 49 | K3 | K23 |
| 26 | 550.949 | 3 | 1650.832 | 1650.837 | -2.76 | [EVK] | alpha-NAC | 11 | 13 | {MTGSTETRQK] | alpha-NAC | 0 | 10 | K3 | T5 |
| 25 | 741.046 | 3 | 2221.123 | 2221.12 | 1.02 | [RKK] | beta-NAC | 31 | 33 | [EADNDIVNAIMSLTm} | alpha-NAC | 181 | 196 | K3 | T14 |
| 25 | 760.728 | 3 | 2280.169 | 2280.169 | -0.03 | [QSRSEK] | alpha-NAC | 55 | 60 | {MmDSKAIAERIK] | beta-NAC | 0 | 12 | S4 | {0 |
| 22 | 618.326 | 3 | 1852.962 | 1852.962 | 0.11 | [VCIRKSK] | alpha-NAC | 80 | 86 | {MTGSTETR] | alpha-NAC | 0 | 8 | K5 | T7 |
| 22 | 478.289 | 3 | 1432.854 | 1432.852 | 0.99 | [QSR] | alpha-NAC | 55 | 57 | [LFSKLGLK] | alpha-NAC | 65 | 72 | S2 | K4 |
| 21 | 442.24 | 3 | 1324.706 | 1324.711 | -3.74 | [VIHK] | beta-NAC | 35 | 38 | [TAAADDK] | beta-NAC | 39 | 45 | K4 | T1 |
| 21 | 741.046 | 3 | 2221.124 | 2221.12 | 1.56 | [RKK] | beta-NAC | 31 | 33 | [EADNDIVNAIMSLTm} | alpha-NAC | 181 | 196 | K3 | T14 |
| 99 | 661.332 | 4 | 2642.307 | 2642.309 | -0.85 | {MTGSTETR] | alpha-NAC | 0 | 8 | [VAEAAGLGDHIDKQAK] | alpha-NAC | 39 | 54 | T2 | K16 |
| 88 | 540.555 | 4 | 2159.197 | 2159.204 | -3.24 | [IGGKGTPR] | beta-NAC | 23 | 30 | [KLQAQQEHVR] | beta-NAC | 13 | 22 | K4 | K1 |
| 66 | 639.869 | 4 | 2556.454 | 2556.462 | -3.07 | [LGPDGK] | beta-NAC | 131 | 136 | [IKKLQAQQEHVRIGGK] | beta-NAC | 11 | 26 | K6 | K2 |
| 66 | 741.912 | 2 | 1482.816 | 1482.816 | 0.15 | [LGPDGK] | beta-NAC | 131 | 136 | [LANNVTK] | beta-NAC | 124 | 130 | K6 | K7 |
| 55 | 471.275 | 2 | 941.542 | 941.541 | 1.07 | [IGGK] | beta-NAC | 23 | 26 | [GTPR] | beta-NAC | 27 | 30 | K4 | T2 |
| 45 | 657.394 | 2 | 1313.781 | 1313.779 | 1.67 | [LGLK] | alpha-NAC | 69 | 72 | [QVTGVSR] | alpha-NAC | 73 | 79 | K4 | T3 |

**Table S9. Intra- TM Im7 cross-links observed for the NAC-TM-Im7 complex**

| Score | m/z | z | M+H+ | Calculated (Da) | Deviation (Da) | Peptide 1 | Protein 1 | From | To | Peptide 2 | Protein 2 | From | To | Site 1 | Site 2 |
| --- | --- | --- | --- | --- | --- | --- | --- | --- | --- | --- | --- | --- | --- | --- | --- |
| 53 | 914.137 | 3 | 2740.396 | 2740.393 | 1.31 | [EIEK] | TM Im7 | 27 | 30 | [ENVAATDDVLDVALEHFVK] | TM Im7 | 31 | 49 | K4 | T6 |
| 159 | 1082.775 | 4 | 4328.077 | 4328.088 | -2.58 | [ENVAATDDVLDVALEHFVK] | TM Im7 | 31 | 49 | [ITEHPDGTDLIYYPSDNR] | TM Im7 | 50 | 67 | K19 | T2 |
| 53 | 969.993 | 2 | 1938.979 | 1938.981 | -0.83 | [DDSPEGIVKEIKEWR] | TM Im7 | 68 | 82 | 1 | intrapeptidal | 0 | 0 | K9 | K12 |

**Table S10. NAC-TM-Im7 cross-links observed for the NAC-TM-Im7 complex**

| Score | m/z | z | M+H+ | Calculated (Da) | Deviation (Da) | Peptide 1 | Protein 1 | From | To | Peptide 2 | Protein 2 | From | To | Site 1 | Site 2 |
| --- | --- | --- | --- | --- | --- | --- | --- | --- | --- | --- | --- | --- | --- | --- | --- |
| 53 | 627.312 | 3 | 1879.922 | 1879.922 | 0.11 | [EIKEWR] | TM Im7 | 77 | 82 | {MTGSTETR] | alpha-NAC | 0 | 8 | K3 | {0 |
| 47 | 808.427 | 3 | 2423.266 | 2423.266 | -0.28 | [DDSPEGIVK] | TM Im7 | 68 | 76 | [LANNVTKLGPDGK] | beta-NAC | 124 | 136 | K9 | K7 |

**References**

1. Whitmore, L., and Wallace, B. A. (2004) DICHROWEB, an online server for protein secondary structure analyses from circular dichroism spectroscopic data. *Nuc. Acid. Res.* **32**, W668-W673

2. Sreerama, N., and Woody, R. W. (2000) Estimation of protein secondary structure from circular dichroism spectra: Comparison of CONTIN, SELCON, and CDSSTR methods with an expanded reference set. *Anal. Biochem.* **287**, 252-260
