# Supplementary material for: Conformational flexibility within the nascent polypeptide–associated complex enables its interactions with structurally diverse client proteins
Source: J Biol Chem. 2018 Apr 12;293(22):8554–68. doi: 10.1074/jbc.RA117.001568 (PMC5986199; doi:10.1074/jbc.RA117.001568)
Supplement: Supporting Information [file supp_RA117.001568_134820_2_supp_114393_p6t8y5.pdf]

Supporting information for conformational flexibility within the nascent polypeptide–associated complex enables its interactions with structurally diverse client proteins

Esther M. Martin[1#], Matthew P. Jackson[1], Martin Gamerdinger[2], Karina Gense[2], Theodoros K. Karamonos[1], Julia R. Humes[1], Elke Deuerling[2], Alison E. Ashcroft[1] and Sheena E. Radford[1*]

From the [1]Astbury Centre for Structural Molecular Biology, School of Molecular and Cellular Biology, Faculty of Biological Sciences, University of Leeds, Leeds, LS2 9JT, UK; [2]Department of Biology, Institute of Molecular Microbiology, University of Konstanz, 78454 Konstanz, Germany

Running title: *Structure and function of NAC*

[#]Present address: MedImmune Ltd, Granta Park, Cambridge, CB21 6GH, UK

*To whom correspondence should be addressed: Sheena E. Radford, Astbury Centre for Structural Molecular Biology, School of Molecular and Cellular Biology, Faculty of Biological Sciences, University of Leeds, Leeds, LS2 9JT; s.e.radford@leeds.ac.uk; +44 113 343 3170.

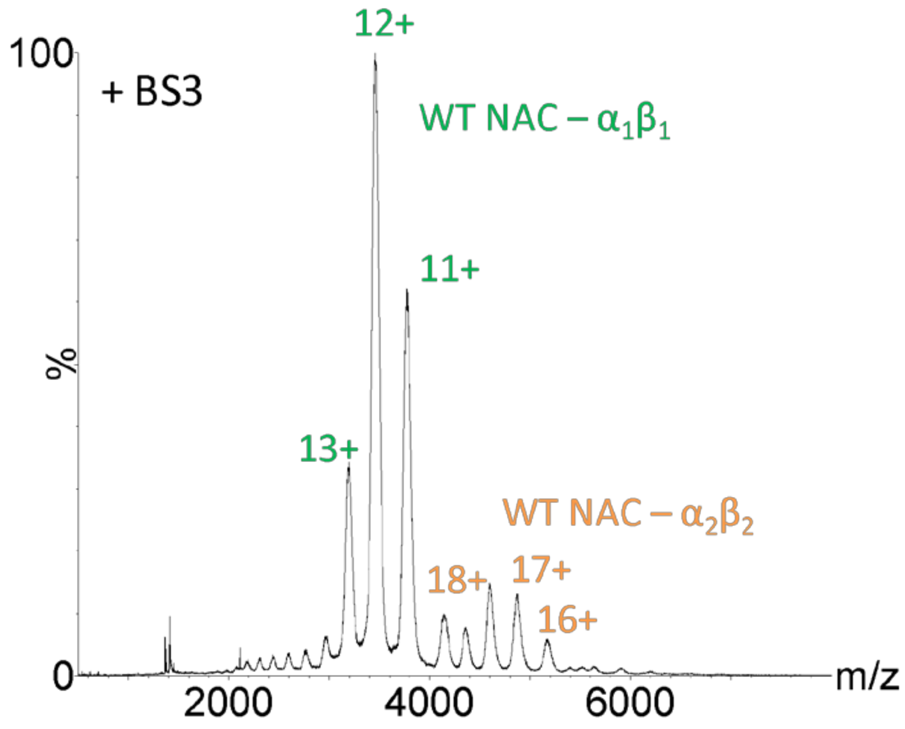

Figure S1: Native ESI-mass spectrum of WT-NAC after cross-linking with BS3. WT-NAC was cross-linked with a 50x molar excess of BS3 at room temperature for 30 min. Following this the protein was buffer exchanged into 100 mM ammonium acetate, pH 6.9 and analysed using native ESI-MS. The spectrum shows a loss in intensity of more highly charged ions (>13^+) generated from more expanded conformers subsequent to chemical cross-linking experiments (compare with Figure 2a).

Supplementary Tables

Table S1: Collisional cross sections of WT NAC and ΔUBA NAC by native mass spectrometry

Native conformation (Å^2)			Extended conformation (Å^2)		
**Charge state**	WT NAC	ΔUBA NAC	**Charge state**	WT NAC	ΔUBA NAC
**10**	-	2693.2	**16**	3896.5, 3767.3	3741.2
**11**	2962.3	2761.7	**17**	4085.7	3886.7, 4205.4
**12**	3048.4	2824.5	**18**	4239.3	4298.1, 4086.2
**13**	3143.7	2925.3	**19**	4475.3, 4630.9	4537.4
**14**	3271.6, 3318.9	3176.3	**20**	4872.7	4776.6
**15**	3767.2, 3628.5	3580.5	**21**	5083.1	4948.5
			**22**	5289.9	5109.5
			**23**	5457.9	5494.4, 5261.6
			**24**	5950.4, 5732.4	-

Table S2: Estimation of NAC secondary structure by CD spectroscopy. Values were estimated by importing the data shown in Figure 2d into DichroWeb (1) and using the CONTIN (2).

**Protein**	**Helix (%)**	**β-strand (%)**	**β-turn (%)**	**Unordered (%)**
WT-NAC	27.5	16.9	22.6	33.0
ΔUBA-NAC	15.1	27.1	21.5	36.0

Table S3. Intra- and inter- NAC cross-links observed for the NAC-α-synuclein complex

Score	m/z	z	M+H+	Calculated (Da)	Deviation (Da)	Peptide 1	Protein 1	From	To	Peptide 2	Protein 2	From	To	Site 1	Site 2
**201**	711.375	3	2132.111	2132.109	0.89	[VAEAAGLGDH IDKQAKQSR]	alpha-NAC	39	57	1	intrapeptidal	0	0	K13	K16
**185**	594.988	3	1782.949	1782.953	-2.39	[QAK]	alpha-NAC	52	54	[VAEAAGLGD HIDK]	alpha-NAC	39	51	K3	K13
**163**	593.648	3	1778.93	1778.928	1.06	[VAEAAGLGDH IDKQAK]	alpha-NAC	39	54	0	dead-end	0	0	K16	x0
**160**	1140.89 6	3	3420.675	3420.686	-3.19	[DDGTVIHFNNP K]	beta-NAC	70	81	[VQTSVPANTF SVTGSADNK]	beta-NAC	82	100	K12	T3
**99**	556.283	3	1666.835	1666.832	2.16	[QKEVK]	alpha-NAC	9	13	{mTGSTETR]	alpha-NAC	0	8	K2	{0
**92**	552.293	3	1654.864	1654.862	1.04	[QKEVK]	alpha-NAC	9	13	{MTGSTETR]	alpha-NAC	0	8	K2	{0
**91**	418.925	3	1254.759	1254.761	-0.86	[SEKK]	alpha-NAC	58	61	[KLFSK]	alpha-NAC	64	68	K3	K1
**86**	474.312	3	1420.922	1420.919	2.35	[KAR]	alpha-NAC	61	63	[LFSKLGLK]	alpha-NAC	65	72	K1	K4
**86**	474.312	3	1420.922	1420.919	2.35	[ARK]	alpha-NAC	62	64	[LFSKLGLK]	alpha-NAC	65	72	K3	K4
**79**	557.624	3	1670.858	1670.857	0.7	[QKEVK]	alpha-NAC	9	13	{mTGSTETR]	alpha-NAC	0	8	K2	{0
**78**	603.654	3	1808.948	1808.947	0.26	[IGGKGTPR]	beta-NAC	23	30	{MTGSTETR]	alpha-NAC	0	8	K4	{0
**76**	640.04	3	1918.106	1918.105	0.86	[SKNILFVINKPD VFK]	alpha-NAC	85	99	0	dead-end	0	0	K2	x0
**73**	462.58	3	1385.725	1385.724	0.86	[KSK]	alpha-NAC	84	86	{MTGSTETR]	alpha-NAC	0	8	K1	{0
**68**	539.314	3	1615.927	1615.928	-0.81	[IKKLQAQQEH VR]	beta-NAC	11	22	1	intrapeptidal	0	0	K2	K3
**68**	823.086	3	2467.242	2467.25	-3.03	[IEDLTQHAQmS AIENLKPTR]	alpha-NAC	114	133	0	dead-end	0	0	K17	x0
**67**	911.502	3	2732.491	2732.49	0.43	[QITEmLPGILN QLGPESLTHLK K]	beta-NAC	101	123	0	dead-end	0	0	T19	x0
**65**	438.95	3	1314.837	1314.841	-2.81	[IKK]	beta-NAC	11	13	[IGGKGTPR]	beta-NAC	23	30	K3	K4
**63**	733.066	3	2197.185	2197.179	2.36	{mTGSTETR]	alpha-NAC	0	8	[LGLKQVTGVS R]	alpha-NAC	69	79	{0	K4
**60**	727.733	3	2181.185	2181.185	0.16	{MTGSTETR]	alpha-NAC	0	8	[LGLKQVTGVS R]	alpha-NAC	69	79	{0	K4
**58**	607.642	3	1820.911	1820.917	-3.1	[IGGKGTPR]	beta-NAC	23	30	{mTGSTETR]	alpha-NAC	0	8	K4	{0
**58**	906.171	3	2716.498	2716.495	0.96	[QITEMLPGILN QLGPESLTHLK K]	beta-NAC	101	123	0	dead-end	0	0	K23	x0
**56**	553.285	3	1657.841	1657.847	-3.32	[KLFSK]	alpha-NAC	64	68	{mTGSTETR]	alpha-NAC	0	8	K1	{0

**56**	911.504	3	2732.496	2732.49	2.18	[QITEmLPGILN QLGPESLTHLK K]	beta-NAC	101	123	0	dead-end	0	0	T19	x0
**55**	731.726	3	2193.164	2193.154	4.5	{mTGSTETR]	alpha-NAC	0	8	[LGLKQVTGVS R]	alpha-NAC	69	79	{0	K4
**55**	472.969	3	1416.893	1416.894	-0.63	[KAR]	alpha-NAC	61	63	[LFSKLGLK]	alpha-NAC	65	72	K1	K8
**55**	472.969	3	1416.893	1416.894	-0.63	[ARK]	alpha-NAC	62	64	[LFSKLGLK]	alpha-NAC	65	72	K3	K8
**55**	676.734	3	2028.188	2028.189	-0.68	[KSKNILFVINKP DVFK]	alpha-NAC	84	99	1	intrapeptidal	0	0	K1	K3
**54**	733.064	3	2197.178	2197.179	-0.47	{mTGSTETR]	alpha-NAC	0	8	[LGLKQVTGVS R]	alpha-NAC	69	79	{0	K4
**54**	823.086	3	2467.244	2467.25	-2.25	[IEDLTQHAQmS AIENLKPTR]	alpha-NAC	114	133	0	dead-end	0	0	K17	x0
**53**	437.61	3	1310.816	1310.815	0.23	[IKK]	beta-NAC	11	13	[IGGKGTPR]	beta-NAC	23	30	K3	K4
**52**	640.041	3	1918.109	1918.105	2.11	[SKNILFVINKPD VFK]	alpha-NAC	85	99	0	dead-end	0	0	K2	x0
**48**	379.914	3	1137.727	1137.729	-1.81	[KAR]	alpha-NAC	61	63	[KLFSK]	alpha-NAC	64	68	K1	K1
**48**	379.914	3	1137.727	1137.729	-1.81	[ARK]	alpha-NAC	62	64	[KLFSK]	alpha-NAC	64	68	K3	K1
**48**	593.647	3	1778.927	1778.928	-0.99	[QAK]	alpha-NAC	52	54	[VAEAAGLGD HIDK]	alpha-NAC	39	51	K3	K13
**48**	593.647	3	1778.927	1778.928	-0.99	[VAEAAGLGDH IDKQAK]	alpha-NAC	39	54	0	dead-end	0	0	K16	x0
**47**	602.312	3	1804.921	1804.922	-0.56	[IGGKGTPR]	beta-NAC	23	30	{MTGSTETR]	alpha-NAC	0	8	K4	{0
**46**	643.01	3	1927.015	1927.01	2.22	[KLANNVTK]	beta-NAC	123	130	{mTGSTETR]	alpha-NAC	0	8	K1	{0
**44**	417.583	3	1250.736	1250.735	0.24	[SEKK]	alpha-NAC	58	61	[KLFSK]	alpha-NAC	64	68	K3	K1
**44**	712.716	3	2136.134	2136.134	0.12	[LGPDGK]	beta-NAC	131	136	{MmDSKAIAE RIK]	beta-NAC	0	12	K6	K12
**44**	712.716	3	2136.134	2136.134	0.12	[LGPDGK]	beta-NAC	131	136	{mMDSKAIAE RIK]	beta-NAC	0	12	K6	K12
**42**	602.313	3	1804.925	1804.922	1.76	[IGGKGTPR]	beta-NAC	23	30	{MTGSTETR]	alpha-NAC	0	8	K4	T2
**41**	676.735	3	2028.191	2028.189	0.86	[KSKNILFVINKP DVFK]	alpha-NAC	84	99	1	intrapeptidal	0	0	K1	K3
**39**	711.375	3	2132.111	2132.109	1.11	[LGPDGK]	beta-NAC	131	136	{MmDSKAIAE RIK]	beta-NAC	0	12	K6	K5
**39**	711.375	3	2132.111	2132.109	1.11	[LGPDGK]	beta-NAC	131	136	{mMDSKAIAE RIK]	beta-NAC	0	12	K6	K5
**39**	602.991	3	1806.957	1806.96	-1.2	[IGGK]	beta-NAC	23	26	[VAEAAGLGD HIDK]	alpha-NAC	39	51	K4	K13
**39**	586.296	3	1756.872	1756.88	-4.34	[QAKQSR]	alpha-NAC	52	57	{mTGSTETR]	alpha-NAC	0	8	K3	T2
**38**	538.986	3	1614.943	1614.936	3.87	[LGPDGK]	beta-NAC	131	136	[KLANNVTK]	beta-NAC	123	130	K6	K8
**36**	604.332	3	1810.98	1810.985	-2.35	[IGGK]	beta-NAC	23	26	[VAEAAGLGD HIDK]	alpha-NAC	39	51	K4	K13
**35**	474.312	3	1420.922	1420.919	2.35	[LGLK]	alpha-NAC	69	72	[ARKLFSK]	alpha-NAC	62	68	K4	K7

**35**	474.312	3	1420.922	1420.919	2.35	[LGLK]	alpha-NAC	69	72	[ARKLFSK]	alpha-NAC	62	68	K4	K7
**33**	551.948	3	1653.83	1653.83	0.3	[VCIRK]	alpha-NAC	80	84	{mTGSTETR]	alpha-NAC	0	8	K5	{0
**32**	731.722	3	2193.153	2193.154	-0.78	{mTGSTETR]	alpha-NAC	0	8	[LGLKQVTGVSR]	alpha-NAC	69	79	{0	T7
**31**	817.757	3	2451.256	2451.255	0.48	[IEDLTQHAQMSAIENLKPTR]	alpha-NAC	114	133	0	dead-end	0	0	K17	x0
**29**	472.969	3	1416.893	1416.894	-0.63	[LGLK]	alpha-NAC	69	72	[ARKLFSK]	alpha-NAC	62	68	K4	K7
**29**	472.969	3	1416.893	1416.894	-0.63	[LGLK]	alpha-NAC	69	72	[ARKLFSK]	alpha-NAC	62	68	K4	K7
**29**	867.145	3	2599.42	2599.413	2.74	[KARKLFSK]	alpha-NAC	61	68	[SPGSDTYIIFGEAK]	alpha-NAC	100	113	K1	T6
**28**	1139.561	3	3416.669	3416.66	2.6	[DDGTVIHFNNPK]	beta-NAC	70	81	[VQTSVPANTFSVTGSADNK]	beta-NAC	82	100	K12	S4
**28**	1139.561	3	3416.669	3416.66	2.6	[DDGTVIHFNNPKVQTSVPANTFSVTGSADNK]	beta-NAC	70	100	0	dead-end	0	0	K12	x0
**27**	538.812	4	2152.225	2152.227	-0.94	[IKK]	beta-NAC	11	13	[VAEAAGLGDHIDKQAK]	alpha-NAC	39	54	K3	K16
**26**	712.716	3	2136.133	2136.134	-0.3	[LGPDGK]	beta-NAC	131	136	{MmDSKAIAERIK]	beta-NAC	0	12	K6	K5
**26**	712.716	3	2136.133	2136.134	-0.3	[LGPDGK]	beta-NAC	131	136	{mMDSKAIAERIK]	beta-NAC	0	12	K6	K5
**26**	1032.54	3	3095.608	3095.619	-3.53	[DIELVISQANTTR]	alpha-NAC	160	172	{MTGSTETRQKEVK]	alpha-NAC	0	13	T12	K10
**23**	602.992	3	1806.961	1806.96	0.85	[IGGK]	beta-NAC	23	26	[VAEAAGLGDHIDK]	alpha-NAC	39	51	K4	K13
**158**	527.739	2	1054.471	1054.472	-0.83	{mTGSTETR]	alpha-NAC	0	8	0	dead-end	0	0	{0	x0
**133**	638.102	4	2549.388	2549.398	-4.16	[IGGKGTPR]	beta-NAC	23	30	[VAEAAGLGDHIDKQAK]	alpha-NAC	39	54	T6	K16
**131**	379.232	2	757.457	757.457	0.08	[NKAIR]	alpha-NAC	173	177	0	dead-end	0	0	K2	x0
**124**	851.991	2	1702.975	1702.978	-1.58	[NILFVINKPDVFK]	alpha-NAC	87	99	0	dead-end	0	0	K8	x0
**118**	471.275	2	941.543	941.541	1.73	[IGGKGTPR]	beta-NAC	23	30	0	dead-end	0	0	T6	x0
**105**	541.562	4	2163.224	2163.229	-2.45	[IGGKGTPR]	beta-NAC	23	30	[KLQAQQEHVR]	beta-NAC	13	22	K4	K1
**103**	473.287	2	945.567	945.567	0.02	[IGGK]	beta-NAC	23	26	[GTPR]	beta-NAC	27	30	K4	T2
**103**	741.911	2	1482.815	1482.816	-1.09	[LANNVTKLGPDGK]	beta-NAC	124	136	0	dead-end	0	0	K7	x0
**102**	522.308	2	1043.611	1043.609	1.06	[KLANNVTK]	beta-NAC	123	130	0	dead-end	0	0	K1	x0
**101**	471.275	2	941.543	941.541	1.73	[IGGK]	beta-NAC	23	26	[GTPR]	beta-NAC	27	30	K4	T2
**87**	657.393	2	1313.779	1313.779	0.1	[LGLKQVTGVSR]	alpha-NAC	69	79	0	dead-end	0	0	K4	x0
**80**	493.797	2	986.587	986.588	-1.06	[KLQSNLK]	beta-NAC	46	52	0	dead-end	0	0	K1	x0
**67**	851.992	2	1702.976	1702.978	-1.23	[NILFVINKPDVFK]	alpha-NAC	87	99	0	dead-end	0	0	K8	x0

**60**	743.923	2	1486.838	1486.841	-2.37	[LGPDGK]	beta-NAC	131	136	[LANNVTK]	beta-NAC	124	130	K6	K7
**58**	569.796	4	2276.161	2276.16	0.55	{mTGSTETR]	alpha-NAC	0	8	[KLQAQQEHVR]	beta-NAC	13	22	{0	K1
**58**	796.952	2	1592.896	1592.901	-2.65	[KLANNVTKLGPDGK]	beta-NAC	123	136	1	intrapeptidal	0	0	K1	K8
**57**	741.911	2	1482.815	1482.816	-1.09	[LGPDGK]	beta-NAC	131	136	[LANNVTK]	beta-NAC	124	130	K6	T6
**55**	696.884	2	1392.76	1392.759	0.74	[KLQAQQEHVR]	beta-NAC	13	22	0	dead-end	0	0	K1	x0
**54**	389.739	2	778.47	778.471	-1.14	[KLFSK]	alpha-NAC	64	68	0	dead-end	0	0	K1	x0
**53**	432.646	5	2159.2	2159.204	-1.91	[IGGKGTPR]	beta-NAC	23	30	[KLQAQQEHVR]	beta-NAC	13	22	K4	K1
**53**	659.405	2	1317.802	1317.804	-1.6	[LGLK]	alpha-NAC	69	72	[QVTGVSR]	alpha-NAC	73	79	K4	T3
**53**	659.405	2	1317.802	1317.804	-1.6	[LGLK]	alpha-NAC	69	72	[QVTGVSR]	alpha-NAC	73	79	K4	T3
**52**	657.393	2	1313.779	1313.779	0.1	[LGLK]	alpha-NAC	69	72	[QVTGVSR]	alpha-NAC	73	79	K4	T3
**52**	657.393	2	1313.779	1313.779	0.1	[LGLK]	alpha-NAC	69	72	[QVTGVSR]	alpha-NAC	73	79	K4	T3
**50**	891.979	2	1782.95	1782.953	-1.73	[QAK]	alpha-NAC	52	54	[VAEAAGLGDHIDK]	alpha-NAC	39	51	K3	K13
**46**	796.95	2	1592.893	1592.901	-4.59	[KLANNVTKLGPDGK]	beta-NAC	123	136	1	intrapeptidal	0	0	K1	T7
**43**	586.377	2	1171.746	1171.745	1.26	[KLFSKLGLK]	alpha-NAC	64	72	1	intrapeptidal	0	0	K1	K5
**42**	661.335	4	2642.318	2642.309	3.46	{MTGSTETR]	alpha-NAC	0	8	[VAEAAGLGDHIDKQAK]	alpha-NAC	39	54	T2	K16
**41**	820.945	2	1640.883	1640.885	-1.74	[TAAADDKKLQSNLK]	beta-NAC	39	52	1	intrapeptidal	0	0	K7	K8
**37**	521.817	4	2084.244	2084.248	-2.22	[IGGKGTPR]	beta-NAC	23	30	[LGLKQVTGVSR]	alpha-NAC	69	79	K4	K4
**31**	854.917	4	3416.645	3416.66	-4.65	[DDGTVIHFNNPKVQTSVPANTFSVTGSADNK]	beta-NAC	70	100	0	dead-end	0	0	K12	x0
**29**	854.917	4	3416.645	3416.66	-4.65	[DDGTVIHFNNPK]	beta-NAC	70	81	[VQTSVPANTFSVTGSADNK]	beta-NAC	82	100	K12	S4
**29**	796.984	2	1592.96	1592.956	2.69	[KLFSK]	alpha-NAC	64	68	[VCIRKSK]	alpha-NAC	80	86	K1	K7
**26**	600.849	4	2400.375	2400.383	-3.62	[KAR]	alpha-NAC	61	63	[KLSVTNIPGIEEVNMIK]	beta-NAC	53	69	K1	K1
**26**	600.849	4	2400.375	2400.383	-3.62	[ARK]	alpha-NAC	62	64	[KLSVTNIPGIEEVNMIK]	beta-NAC	53	69	K3	K1
**26**	855.923	4	3420.67	3420.686	-4.47	[DDGTVIHFNNPK]	beta-NAC	70	81	[VQTSVPANTFSVTGSADNK]	beta-NAC	82	100	K12	T3
**25**	432.646	5	2159.2	2159.204	-1.91	[KLQAQQEHVRIGGKGTPR]	beta-NAC	13	30	0	dead-end	0	0	K1	x0
**25**	662.338	4	2646.331	2646.334	-1.34	{MTGSTETR]	alpha-NAC	0	8	[VAEAAGLGDHIDKQAK]	alpha-NAC	39	54	{0	K16
**25**	844.435	4	3374.717	3374.705	3.57	{MmDSK]	beta-NAC	0	5	{MMDSKAIAERIKKLQAQQEHVR]	beta-NAC	0	22	{0	K5

**23**	600.849	4	2400.375	2400.383	-3.62	[KARK]	alpha-NAC	61	64	[LSVTNIPGIEE VNMIK]	beta-NAC	54	69	K4	T4
**22**	403.487	4	1610.926	1610.934	-4.73	[GTPRRK]	beta-NAC	27	32	[LANNVTK]	beta-NAC	124	130	K6	K7
**20**	855.925	4	3420.679	3420.686	-1.81	[DDGTVIHFNNP K]	beta-NAC	70	81	[VQTSVPANTF SVTGSADNK]	beta-NAC	82	100	K12	T9
**19**	433.452	5	2163.229	2163.229	0.01	[IGGKGTPR]	beta-NAC	23	30	[KLQAQQEHV R]	beta-NAC	13	22	K4	K1
**18**	404.491	4	1614.94	1614.936	2.39	[LGPDGK]	beta-NAC	131	136	[KLANNVTK]	beta-NAC	123	130	K6	K8
**18**	662.338	4	2646.331	2646.326	1.83	[LGPDGK]	beta-NAC	131	136	[VQTSVPANTF SVTGSADNK]	beta-NAC	82	100	K6	K19

Table S4. Intra- α-synuclein cross-links observed for the NAC-α-synuclein complex

Score	m/z	z	M+H+	Calculated (Da)	Deviation (Da)	Peptide 1	Protein 1	From	To	Peptide 2	Protein 2	From	To	Site 1	Site 2
**144**	668.872	2	1336.736	1336.736	0.05	[EGVLYVGSKTK]	alpha-synuclein	35	45	0	dead-end	0	0	T10	x0
**125**	865.807	3	2595.407	2595.403	1.62	[EGVLYVGSKTKEGVVHGVATVAEK]	alpha-synuclein	35	58	1	intrapeptidal	0	0	K9	T10
**85**	505.611	3	1514.82	1514.826	-4.2	[GLSKAK]	alpha-synuclein	7	12	{MDVFMK]	alpha-synuclein	0	6	K6	{0
**82**	771.758	3	2313.26	2313.266	-2.45	[TKEQVTNVGGAVVTGVTAVAQK]	alpha-synuclein	59	80	0	dead-end	0	0	K2	x0
**80**	893.736	4	3571.922	3571.933	-3.08	[EGVVHGVATVAEKTKEQVTNVGGAVVTGVTAVAQK]	alpha-synuclein	46	80	1	intrapeptidal	0	0	K15	T14
**79**	608.333	2	1215.658	1215.658	0.31	[TKQGVAEAAGK]	alpha-synuclein	22	32	0	dead-end	0	0	K2	x0
**74**	798.454	2	1595.901	1595.9	0.39	[GLSKAKEGVVAAAEK]	alpha-synuclein	7	21	1	intrapeptidal	0	0	K4	K6
**64**	560.978	3	1680.918	1680.917	0.83	[TKEGVVHGVATVAEK]	alpha-synuclein	44	58	0	dead-end	0	0	K2	x0
**64**	684.703	3	2052.094	2052.097	-1.32	[EGVVAAAEKTKQGVAEAAGK]	alpha-synuclein	13	32	1	intrapeptidal	0	0	K11	T10
**63**	672.684	3	2016.036	2016.033	1.47	{mDVFmK]	alpha-synuclein	0	6	[AKEGVVAAAEK]	alpha-synuclein	11	21	{0	K2
**59**	422.741	2	844.476	844.477	-2.15	[KDQLGK]	alpha-synuclein	97	102	0	dead-end	0	0	K1	x0
**57**	614.842	2	1228.677	1228.678	-1.48	[AKEGVVAAAEK]	alpha-synuclein	11	21	0	dead-end	0	0	K2	x0
**55**	569.07	4	2273.26	2273.265	-2.25	[TKQGVAEAAGK]	alpha-synuclein	22	32	[AKEGVVAAAEK]	alpha-synuclein	11	21	K2	K2
**51**	881.98	2	1762.952	1762.959	-3.59	[TVEGAGSIAAATGFVKK]	alpha-synuclein	81	97	0	dead-end	0	0	T12	x0
**50**	560.977	3	1680.918	1680.917	0.51	[TKEGVVHGVATVAEK]	alpha-synuclein	44	58	0	dead-end	0	0	T1	x0
**49**	672.683	3	2016.035	2016.033	1.17	{mDVFmK]	alpha-synuclein	0	6	[AKEGVVAAAEK]	alpha-synuclein	11	21	{0	K2
**48**	840.96	2	1680.913	1680.917	-2.23	[TKEGVVHGVATVAEK]	alpha-synuclein	44	58	0	dead-end	0	0	T1	x0
**48**	1026.55	2	2052.094	2052.097	-1.19	[EGVVAAAEKTKQGVAEAAGK]	alpha-synuclein	13	32	1	intrapeptidal	0	0	K9	K11
**46**	655.33	2	1309.652	1309.653	-0.71	{mDVFMKGLSK]	alpha-synuclein	0	10	1	intrapeptidal	0	0	{0	K6

**46**	655.33	2	1309.652	1309.653	-0.71	{MDVFmKGLSK]	alpha-synuclein	0	10	1	intrapeptidal	0	0	{0	K6
**45**	712.403	4	2846.591	2846.581	3.31	[TKEGVLYVGSK]	alpha-synuclein	33	43	[TKEGVVHGVATVAEK]	alpha-synuclein	44	58	T1	K2
**45**	663.329	2	1325.651	1325.648	2.6	{mDVFmKGLSK]	alpha-synuclein	0	10	1	intrapeptidal	0	0	{0	K6
**45**	671.342	3	2012.011	2012.008	1.48	{mDVFmK]	alpha-synuclein	0	6	[AKEGVVAAAEK]	alpha-synuclein	11	21	{0	K2
**41**	569.07	4	2273.26	2273.265	-2.25	[QGVAEAAGKTK]	alpha-synuclein	24	34	[AKEGVVAAAEK]	alpha-synuclein	11	21	K11	K2
**39**	712.403	4	2846.591	2846.581	3.31	[TKEGVLYVGSK]	alpha-synuclein	33	43	[EGVVHGVATVAEKTK]	alpha-synuclein	46	60	T1	K15
**38**	568.065	4	2269.237	2269.24	-1.13	[TKQGVAEAAGK]	alpha-synuclein	22	32	[AKEGVVAAAEK]	alpha-synuclein	11	21	K2	K2
**37**	629.849	2	1258.691	1258.689	1.76	[EGVVAAAEKTK]	alpha-synuclein	13	23	0	dead-end	0	0	K11	x0
**35**	671.338	3	2011.999	2012.008	-4.34	{mDVFmK]	alpha-synuclein	0	6	[AKEGVVAAAEK]	alpha-synuclein	11	21	K6	K2
**35**	516.275	3	1546.814	1546.816	-1.35	[GLSKAK]	alpha-synuclein	7	12	{mDVFmK]	alpha-synuclein	0	6	K6	{0
**35**	532.637	3	1595.897	1595.9	-2.04	[GLSKAKEGVVAAAEK]	alpha-synuclein	7	21	1	intrapeptidal	0	0	K4	K6
**34**	616.366	3	1847.082	1847.079	2.06	[GLSKAK]	alpha-synuclein	7	12	[EGVVAAAEKTK]	alpha-synuclein	13	23	K6	K11
**32**	514.933	3	1542.786	1542.791	-3.22	[GLSKAK]	alpha-synuclein	7	12	{mDVFmK]	alpha-synuclein	0	6	K6	{0
**30**	720.409	2	1439.811	1439.81	0.28	[AKEGVVAAAEKTK]	alpha-synuclein	11	23	1	intrapeptidal	0	0	K2	T12
**30**	878.978	2	1756.948	1756.944	2.33	[DQLGK]	alpha-synuclein	98	102	[QGVAEAAGKTK]	alpha-synuclein	24	34	K5	K11
**30**	758.426	3	2273.262	2273.265	-1.25	[TKQGVAEAAGK]	alpha-synuclein	22	32	[AKEGVVAAAEK]	alpha-synuclein	11	21	T1	K11
**30**	757.084	3	2269.238	2269.233	2.37	[EGVLYVGSK]	alpha-synuclein	35	43	[TKEGVLYVGSK]	alpha-synuclein	33	43	Y5	K11
**30**	514.933	3	1542.785	1542.791	-3.41	[GLSKAK]	alpha-synuclein	7	12	{mDVFmK]	alpha-synuclein	0	6	K4	{0
**30**	1032.214	3	3094.627	3094.631	-1.04	[TVEGAGSIAAATGFVK]	alpha-synuclein	81	96	[TVEGAGSIAAATGFVK]	alpha-synuclein	81	96	K16	S7
**28**	568.065	4	2269.237	2269.24	-1.13	[QGVAEAAGKTK]	alpha-synuclein	24	34	[AKEGVVAAAEK]	alpha-synuclein	11	21	K11	K2
**23**	538.812	4	2152.225	2152.216	4.28	[GLSK]	alpha-synuclein	7	10	[TVEGAGSIAAATGFVKK]	alpha-synuclein	81	97	K4	K17
**22**	595.079	4	2377.296	2377.297	-0.73	[TKQGVAEAAGK]	alpha-synuclein	22	32	[TKEGVLYVGSK]	alpha-synuclein	33	43	K2	K2
**19**	595.079	4	2377.296	2377.297	-0.73	[TKQGVAEAAGK]	alpha-synuclein	22	32	[EGVLYVGSKTK]	alpha-synuclein	35	45	K2	K11

Table S5. NAC-α-synuclein cross-links observed for the NAC-α-synuclein complex

Score	m/z	z	M+H+	Calculated (Da)	Deviation (Da)	Peptide 1	Protein 1	From	To	Peptide 2	Protein 2	From	To	Site 1	Site 2
**53**	865.807	3	2595.407	2595.418	-4.13	[EGVLYVGSK]	alpha-synuclein	35	43	[TAAADDKKLQSNLK]	beta-NAC	39	52	Y5	K8
**51**	404.491	4	1614.94	1614.948	-4.55	[KDQLGK]	alpha-synuclein	97	102	[IGGKGTPR]	beta-NAC	23	30	K1	K4
**44**	1037.54	3	3110.607	3110.604	1.01	[TVEGAGSIAAATGFVK]	alpha-synuclein	81	96	{MTGSTETRQKEVK]	alpha-NAC	0	13	S7	K10
**38**	722.42	2	1443.833	1443.835	-1.85	[VCIRK]	alpha-NAC	80	84	[KDQLGK]	alpha-synuclein	97	102	K5	K1
**36**	586.377	2	1171.746	1171.752	-4.88	[RKK]	beta-NAC	31	33	[GLSKAK]	alpha-synuclein	7	12	K2	K4
**35**	516.304	3	1546.897	1546.895	0.9	[DQLGK]	alpha-synuclein	98	102	[ARKLFSK]	alpha-NAC	62	68	K5	S6
**33**	594.99	3	1782.954	1782.96	-3	[LGPDGK]	beta-NAC	131	136	[QGVAEAAGKTK]	alpha-synuclein	24	34	K6	K11
**33**	594.99	3	1782.954	1782.96	-3	[LGPDGK]	beta-NAC	131	136	[TKQGVAEAAGK]	alpha-synuclein	22	32	K6	K11
**33**	634.114	4	2533.433	2533.429	1.92	[IGGKGTPR]	beta-NAC	23	30	[TVEGAGSIAAATGFVKK]	alpha-synuclein	81	97	K4	K17
**32**	712.716	3	2136.134	2136.13	2.25	[QGVAEAAGK]	alpha-synuclein	24	32	[VIHKTAAADDK]	beta-NAC	35	45	K9	K11
**32**	798.134	4	3189.515	3189.516	-0.07	{MDVFMKGLSK]	alpha-synuclein	0	10	[GEDEDVPELVGDFDAASK]	beta-NAC	137	154	K6	K18
**28**	538.986	3	1614.943	1614.948	-3.09	[KDQLGK]	alpha-synuclein	97	102	[IGGKGTPR]	beta-NAC	23	30	K6	T6
**26**	741.911	2	1482.815	1482.817	-1.67	[RKKK]	beta-NAC	31	34	{MDVFmK]	alpha-synuclein	0	6	K3	{0
**18**	1063.95	5	5315.719	5315.697	4.25	[EGVVAAAEKTKQGVAEAAGK]	alpha-synuclein	13	32	[DDGTVIHFNNPKVQTSVPANTFSVTGSADNK]	beta-NAC	70	100	K20	K31

Table S6. Intra- and inter- NAC cross-links observed for the NAC-WT Im7 complex

Score	m/z	z	M+H+	Calculated (Da)	Deviation (Da)	Peptide 1	Protein 1	From	To	Peptide 2	Protein 2	From	To	Site 1	Site 2
**120**	550.951	3	1650.838	1650.837	0.82	[QKEVK]	alpha-NAC	9	13	{MTGSTETR]	alpha-NAC	0	8	K2	{0
**105**	607.643	3	1820.916	1820.917	-0.79	[IGGKGTPR]	beta-NAC	23	30	{mTGSTETR]	alpha-NAC	0	8	K4	{0
**101**	636.337	3	1906.996	1906.99	3.09	{MTGSTETR]	alpha-NAC	0	8	[KLANNVTK]	beta-NAC	123	130	{0	T7
**85**	602.311	3	1804.918	1804.922	-2.56	[IGGKGTPR]	beta-NAC	23	30	{MTGSTETR]	alpha-NAC	0	8	K4	{0
**77**	556.281	3	1666.83	1666.832	-1.19	[QKEVK]	alpha-NAC	9	13	{mTGSTETR]	alpha-NAC	0	8	K2	{0
**76**	641.667	3	1922.987	1922.985	0.74	[KLANNVTK]	beta-NAC	123	130	{mTGSTETR]	alpha-NAC	0	8	K1	{0
**70**	618.962	3	1854.873	1854.875	-1.13	[TAAADDKK]	beta-NAC	39	46	{mTGSTETR]	alpha-NAC	0	8	K7	{0
**69**	731.725	3	2193.161	2193.154	3	{mTGSTETR]	alpha-NAC	0	8	[LGLKQVTGVSR]	alpha-NAC	69	79	{0	S4
**63**	607.643	3	1820.914	1820.917	-1.45	[IGGKGTPR]	beta-NAC	23	30	{mTGSTETR]	alpha-NAC	0	8	K4	{0
**60**	613.633	3	1838.886	1838.88	2.99	[TAAADDKK]	beta-NAC	39	46	{MTGSTETR]	alpha-NAC	0	8	K7	{0
**60**	970.828	3	2910.471	2910.485	-4.97	{mTGSTETR]	alpha-NAC	0	8	[LQAQQEHVRIGGKGTPR]	beta-NAC	14	30	T2	K13
**60**	584.955	3	1752.849	1752.854	-2.9	[QAKQSR]	alpha-NAC	52	57	{mTGSTETR]	alpha-NAC	0	8	K3	T2
**53**	602.311	3	1804.918	1804.922	-2.56	[IGGKGTPR]	beta-NAC	23	30	{MTGSTETR]	alpha-NAC	0	8	K4	T2
**49**	613.632	3	1838.88	1838.88	0.2	[TAAADDKK]	beta-NAC	39	46	{MTGSTETR]	alpha-NAC	0	8	K7	{0
**42**	881.444	3	2642.317	2642.309	3.02	{MTGSTETR]	alpha-NAC	0	8	[VAEAAGLGDHIDKQAK]	alpha-NAC	39	54	T2	K13
**131**	531.292	4	2122.146	2122.15	-2.06	[KSK]	alpha-NAC	84	86	[VAEAAGLGDHIDKQAK]	alpha-NAC	39	54	K3	K16
**124**	540.554	4	2159.194	2159.204	-4.9	[IGGKGTPR]	beta-NAC	23	30	[KLQAQQEHVR]	beta-NAC	13	22	K4	K1
**123**	537.804	4	2148.195	2148.202	-3.52	[IKK]	beta-NAC	11	13	[VAEAAGLGDHIDKQAK]	alpha-NAC	39	54	K3	K16
**115**	471.272	2	941.537	941.541	-4.38	[IGGK]	beta-NAC	23	26	[GTPR]	beta-NAC	27	30	K4	T2
**81**	428.926	3	1284.762	1284.763	-0.98	[KSK]	alpha-NAC	84	86	[IGGKGTPR]	beta-NAC	23	30	K3	K4
**77**	741.911	2	1482.815	1482.816	-0.75	[LGPDGK]	beta-NAC	131	136	[LANNVTK]	beta-NAC	124	130	K6	K6
**66**	759.768	5	3794.812	3794.824	-3.27	[ARKLFSK]	alpha-NAC	62	68	[GEDEDVPELVGDFDAASKNETKADEQ}	beta-NAC	137	163	S6	K18
**60**	602.992	3	1806.96	1806.96	0.36	[IGGK]	beta-NAC	23	26	[VAEAAGLGDHIDK]	alpha-NAC	39	51	K4	K13
**59**	657.393	2	1313.778	1313.779	-0.46	[LGLK]	alpha-NAC	69	72	[QVTGVSR]	alpha-NAC	73	79	K4	T3

**56**	471.274	2	941.54	941.541	-1.42	[IGGK]	beta-NAC	23	26	[GTPR]	beta-NAC	27	30	K4	T2
**56**	854.918	4	3416.648	3416.66	-3.55	[DDGTVIHFNNPK]	beta-NAC	70	81	[VQTSVPANTFSVTGSADNK]	beta-NAC	82	100	K12	S4
**52**	432.646	5	2159.199	2159.204	-2.6	[IGGKGTPR]	beta-NAC	23	30	[KLQAQQEHVR]	beta-NAC	13	22	K4	K1
**49**	889.969	2	1778.931	1778.928	1.47	[QAK]	alpha-NAC	52	54	[VAEAAGLGDHIDK]	alpha-NAC	39	51	K3	K13
**47**	537.804	4	2148.195	2148.202	-3.52	[LGPDGK]	beta-NAC	131	136	[KKVIHKTAAADDK]	beta-NAC	33	45	K6	K13
**47**	639.869	4	2556.454	2556.462	-3.07	[LGPDGK]	beta-NAC	131	136	[IKKLQAQQEHVRIGGK]	beta-NAC	11	26	K6	K3
**46**	889.968	2	1778.928	1778.928	-0.16	[QAK]	alpha-NAC	52	54	[VAEAAGLGDHIDK]	alpha-NAC	39	51	K3	K13
**39**	428.926	3	1284.762	1284.763	-0.98	[LGLK]	alpha-NAC	69	72	[QAKQSR]	alpha-NAC	52	57	K4	S5
**37**	463.777	4	1852.087	1852.09	-1.58	[IKK]	beta-NAC	11	13	[LANNVTKLGPDGK]	beta-NAC	124	136	K3	K7
**37**	854.92	4	3416.659	3416.66	-0.47	[DDGTVIHFNNPK]	beta-NAC	70	81	[VQTSVPANTFSVTGSADNK]	beta-NAC	82	100	K12	T3
**34**	871.472	2	1741.937	1741.944	-4.34	[IGGKGTPR]	beta-NAC	23	30	[TAAADDKK]	beta-NAC	39	46	K4	K7
**33**	687.867	2	1374.728	1374.722	3.78	[NETK]	beta-NAC	155	158	[QVTGVSR]	alpha-NAC	73	79	K4	S6
**32**	530.311	2	1059.615	1059.616	-1.09	[RKK]	beta-NAC	31	33	[NETK]	beta-NAC	155	158	K2	K4
**31**	534.293	4	2134.151	2134.15	0.15	[IGGK]	beta-NAC	23	26	[VAEAAGLGDHIDKQAK]	alpha-NAC	39	54	K4	K16
**30**	430.445	5	2148.197	2148.202	-2.61	[IKK]	beta-NAC	11	13	[VAEAAGLGDHIDKQAK]	alpha-NAC	39	54	K3	K13
**30**	531.333	2	1061.658	1061.661	-2.62	[LGLK]	alpha-NAC	69	72	[LFSK]	alpha-NAC	65	68	K4	K4
**25**	512.1	5	2556.471	2556.462	3.66	[LGPDGK]	beta-NAC	131	136	[IKKLQAQQEHVRIGGK]	beta-NAC	11	26	K6	K16
**24**	395.981	4	1580.903	1580.901	1.4	[TAAADDK]	beta-NAC	39	45	[KKVIHK]	beta-NAC	33	38	K7	K6
**24**	395.981	4	1580.903	1580.901	1.4	[KVIHK]	beta-NAC	34	38	[TAAADDKK]	beta-NAC	39	46	K5	K8
**24**	537.805	4	2148.199	2148.202	-1.73	[IKK]	beta-NAC	11	13	[VAEAAGLGDHIDKQAK]	alpha-NAC	39	54	K3	K16

Table S7. NAC-WT Im7 cross-links observed for the NAC-WT Im7 complex

Score	m/z	z	M+H+	Calculated (Da)	Deviation (Da)	Peptide 1	Protein 1	From	To	Peptide 2	Protein 2	From	To	Site 1	Site 2
**56**	627.31	3	1879.92	1879.92	-3.24	[EIKEWR]	WT Im7	77	82	{MTGSTETR]	alpha-NAC	0	8	K3	{0
**98**	789.055	3	2365.15	2365.14	3.02	{mTGSTETR]	alpha-NAC	0	8	[DDSPEGIVKEI K]	WT Im7	68	79	S4	K9

Table S8. Intra- and inter- NAC cross-links observed for the NAC-TM-Im7 complex

Score	m/z	z	M+H+	Calculated (Da)	Deviation (Da)	Peptide 1	Protein 1	From	To	Peptide 2	Protein 2	From	To	Site 1	Site 2
**231**	593.647	3	1778.927	1778.928	-0.77	[QAK]	alpha-NAC	52	54	[VAEAAGLGDH IDK]	alpha-NAC	39	51	K3	K13
**113**	1139.55 6	3	3416.654	3416.66	-1.79	[DDGTVIHFNNP K]	beta-NAC	70	81	[VQTSVPANTFS VTGSADNK]	beta-NAC	82	100	K12	T3
**96**	504.252	3	1510.743	1510.742	0.67	[SEKK]	alpha-NAC	58	61	{MTGSTETR]	alpha-NAC	0	8	K3	{0
**93**	550.949	3	1650.832	1650.837	-2.76	[QKEVK]	alpha-NAC	9	13	{MTGSTETR]	alpha-NAC	0	8	K2	{0
**68**	602.311	3	1804.917	1804.922	-2.73	[IGGKGTPR]	beta-NAC	23	30	{MTGSTETR]	alpha-NAC	0	8	K4	{0
**65**	437.609	3	1310.813	1310.815	-1.6	[IKK]	beta-NAC	11	13	[IGGKGTPR]	beta-NAC	23	30	K3	K4
**60**	461.239	3	1381.701	1381.699	1.51	[KSK]	alpha-NAC	84	86	{MTGSTETR]	alpha-NAC	0	8	K1	{0
**55**	602.313	3	1804.925	1804.922	1.43	[IGGKGTPR]	beta-NAC	23	30	{MTGSTETR]	alpha-NAC	0	8	K4	{0
**52**	556.279	3	1666.823	1666.832	-4.97	[QKEVK]	alpha-NAC	9	13	{mTGSTETR]	alpha-NAC	0	8	K2	{0
**52**	726.393	3	2177.163	2177.159	1.8	{MTGSTETR]	alpha-NAC	0	8	[LGLKQVTGVS R]	alpha-NAC	69	79	{0	K4
**45**	607.645	3	1820.921	1820.917	2	[IGGKGTPR]	beta-NAC	23	30	{mTGSTETR]	alpha-NAC	0	8	K4	{0
**44**	711.374	3	2132.107	2132.109	-1	[LGPDGK]	beta-NAC	131	136	{mMDSKAIAERI K]	beta-NAC	0	12	K6	K12
**37**	579.626	3	1736.862	1736.86	1.54	[QAKQSR]	alpha-NAC	52	57	{MTGSTETR]	alpha-NAC	0	8	K3	{0
**33**	628.005	3	1882.001	1881.992	4.8	[IGGKGTPR]	beta-NAC	23	30	[DDSPEGIVK]	TM Im7	68	76	K4	K9
**30**	879.434	3	2636.288	2636.291	-0.85	[QSRSEKK]	alpha-NAC	55	61	[EADNDIVNAIM SLTM}	alpha-NAC	181	196	K6	T14
**29**	803.757	3	2409.257	2409.266	-3.89	{mmDSK]	beta-NAC	0	5	[LGLKQVTGVS RVCIR]	alpha-NAC	69	83	{0	T7
**28**	1032.87 2	3	3096.602	3096.599	1.08	[EVK]	alpha-NAC	11	13	[EIEKENVAATD DVLDVALEHFV K]	TM Im7	27	49	K3	K23
**26**	550.949	3	1650.832	1650.837	-2.76	[EVK]	alpha-NAC	11	13	{MTGSTETRQK]	alpha-NAC	0	10	K3	T5
**25**	741.046	3	2221.123	2221.12	1.02	[RKK]	beta-NAC	31	33	[EADNDIVNAIM SLTm}	alpha-NAC	181	196	K3	T14
**25**	760.728	3	2280.169	2280.169	-0.03	[QSRSEK]	alpha-NAC	55	60	{MmDSKAIAERI K]	beta-NAC	0	12	S4	{0
**22**	618.326	3	1852.962	1852.962	0.11	[VCIRKSK]	alpha-NAC	80	86	{MTGSTETR]	alpha-NAC	0	8	K5	T7
**22**	478.289	3	1432.854	1432.852	0.99	[QSR]	alpha-NAC	55	57	[LFSKLGLK]	alpha-NAC	65	72	S2	K4
**21**	442.24	3	1324.706	1324.711	-3.74	[VIHK]	beta-NAC	35	38	[TAAADDK]	beta-NAC	39	45	K4	T1
**21**	741.046	3	2221.124	2221.12	1.56	[RKK]	beta-NAC	31	33	[EADNDIVNAIM SLTm}	alpha-NAC	181	196	K3	T14
**99**	661.332	4	2642.307	2642.309	-0.85	{MTGSTETR]	alpha-NAC	0	8	[VAEAAGLGDH IDKQAK]	alpha-NAC	39	54	T2	K16

**88**	540.555	4	2159.197	2159.204	-3.24	[IGGKGTPR]	beta-NAC	23	30	[KLQAQQEHVR]	beta-NAC	13	22	K4	K1
**66**	639.869	4	2556.454	2556.462	-3.07	[LGPDGK]	beta-NAC	131	136	[IKKLQAQQEH VRIGGK]	beta-NAC	11	26	K6	K2
**66**	741.912	2	1482.816	1482.816	0.15	[LGPDGK]	beta-NAC	131	136	[LANNVTK]	beta-NAC	124	130	K6	K7
**55**	471.275	2	941.542	941.541	1.07	[IGGK]	beta-NAC	23	26	[GTPR]	beta-NAC	27	30	K4	T2
**45**	657.394	2	1313.781	1313.779	1.67	[LGLK]	alpha-NAC	69	72	[QVTGVSR]	alpha-NAC	73	79	K4	T3

Table S9. Intra- TM Im7 cross-links observed for the NAC-TM-Im7 complex

Score	m/z	z	M+H+	Calculated (Da)	Deviation (Da)	Peptide 1	Protein 1	From	To	Peptide 2	Protein 2	From	To	Site 1	Site 2
**53**	914.137	3	2740.396	2740.393	1.31	[EIEK]	TM Im7	27	30	[ENVAATDDVL DVALEHFVK]	TM Im7	31	49	K4	T6
**159**	1082.77 5	4	4328.077	4328.088	-2.58	[ENVAATDDVL DVALEHFVK]	TM Im7	31	49	[ITEHPDGTDLIY YPSDNR]	TM Im7	50	67	K19	T2
**53**	969.993	2	1938.979	1938.981	-0.83	[DDSPEGIVKEI KEWR]	TM Im7	68	82	1	intrapeptidal	0	0	K9	K12

Table S10. NAC-TM-Im7 cross-links observed for the NAC-TM-Im7 complex

Score	m/z	z	M+H+	Calculated (Da)	Deviation (Da)	Peptide 1	Protein 1	From	To	Peptide 2	Protein 2	From	To	Site 1	Site 2
**53**	627.312	3	1879.922	1879.922	0.11	[EIKEWR]	TM Im7	77	82	{MTGSTETR]	alpha-NAC	0	8	K3	{0
**47**	808.427	3	2423.266	2423.266	-0.28	[DDSPEGIVK]	TM Im7	68	76	[LANNVTKLGPDG K]	beta-NAC	124	136	K9	K7

References

1. Whitmore, L., and Wallace, B. A. (2004) DICHROWEB, an online server for protein secondary structure analyses from circular dichroism spectroscopic data. *Nuc. Acid. Res.* **32**, W668-W673
2. Sreerama, N., and Woody, R. W. (2000) Estimation of protein secondary structure from circular dichroism spectra: Comparison of CONTIN, SELCON, and CDSSTR methods with an expanded reference set. *Anal. Biochem.* **287**, 252-260
